# Fine Mapping of the *Bsr1* Barley Stripe Mosaic Virus Resistance Gene in the Model Grass *Brachypodium distachyon*

**DOI:** 10.1371/journal.pone.0038333

**Published:** 2012-06-04

**Authors:** Yu Cui, Mi Yeon Lee, Naxin Huo, Jennifer Bragg, Lijie Yan, Cheng Yuan, Cui Li, Sara J. Holditch, Jingzhong Xie, Ming-Cheng Luo, Dawei Li, Jialin Yu, Joel Martin, Wendy Schackwitz, Yong Qiang Gu, John P. Vogel, Andrew O. Jackson, Zhiyong Liu, David F. Garvin

**Affiliations:** 1 State Key Laboratory of Agro-Biotechnology, China Agricultural University, Beijing, China; 2 Department of Plant and Microbiology, University of California, Berkeley, California, United States of America; 3 USDA-ARS Western Regional Research Center, Albany, California, United States of America; 4 Department of Plant Sciences, University of California Davis, Davis, California, United States of America; 5 US DOE Joint Genome Institute, Walnut Creek, California, United States of America; 6 USDA-ARS Plant Science Research Unit and Department of Agronomy and Plant Genetics, University of Minnesota, St. Paul, Minnesota, United States of America; University of Massachusetts Amherst, United States of America

## Abstract

The ND18 strain of *Barley stripe mosaic virus* (BSMV) infects several lines of *Brachypodium distachyon*, a recently developed model system for genomics research in cereals. Among the inbred lines tested, Bd3-1 is highly resistant at 20 to 25°C, whereas Bd21 is susceptible and infection results in an intense mosaic phenotype accompanied by high levels of replicating virus. We generated an F_6∶7_ recombinant inbred line (RIL) population from a cross between Bd3-1 and Bd21 and used the RILs, and an F_2_ population of a second Bd21 × Bd3-1 cross to evaluate the inheritance of resistance. The results indicate that resistance segregates as expected for a single dominant gene, which we have designated *Barley stripe mosaic virus resistance 1* (*Bsr1*). We constructed a genetic linkage map of the RIL population using SNP markers to map this gene to within 705 Kb of the distal end of the top of chromosome 3. Additional CAPS and Indel markers were used to fine map *Bsr1* to a 23 Kb interval containing five putative genes. Our study demonstrates the power of using RILs to rapidly map the genetic determinants of BSMV resistance in Brachypodium. Moreover, the RILs and their associated genetic map, when combined with the complete genomic sequence of Brachypodium, provide new resources for genetic analyses of many other traits.

## Introduction

*Barley stripe mosaic virus* (BSMV) is a positive strand tripartite RNA virus whose native host is barley (*Hordeum vulgare* L.), in which serious yield losses can occur [1], [2]. BSMV was originally thought to be restricted to barley and occasionally to wheat in the field, but over the past 25 years, the virus has been shown to occur naturally in field infections of several other cereals [3]–[5]. In addition, BSMV has an extensive experimental host range that includes a wide range of cereals and other grasses plus several dicot species [3]. BSMV disease symptoms can usually be observed 4–7 days after inoculation of susceptible plants. A variety of phenotypic responses ranging from mosaic symptoms, stripes, chlorotic spots and local lesions, to stunting and necrosis have been observed in different hosts. However, plants typically recover within two weeks after symptom appearance and enter a chronic stage of infection characterized by milder symptoms. We have previously conducted reverse genetic analyses of the BSMV Type and ND18 strains that have revealed the molecular determinants of several of these disease phenotypes [6]–[9].

BSMV is of major economic importance to barley cultivation worldwide, and substantial yield losses have been documented in the Northern United States and Canada, where barley is one of the main crops [1], [3]. Estimated yield losses due to BSMV were as large as 25 to 30% in naturally infected field plots in Montana and North Dakota in the 1950’s to 1970’s [10]–[13]. Seed transmission of the virus to progeny and mechanical transmission between plants growing in close proximity to each other in the field are required for virus survival [3]. These transmission properties have permitted substantial inroads in eradicating infections by rogueing infected plants, screening to eliminate virus-infested seed from commercial stocks and incorporation of resistance genes into cultivars [2]. During the past two decades, major progress has been made towards the understanding of infection processes of BSMV, and the virus has become a model for studies of pathogenesis and movement [4], [14], [15]. In contrast, relatively little is known about the fundamental basis of BSMV resistance in host plants [16].

BSMV resistance in barley has been known for many years and the inheritance of resistance has been the subject of several studies [16]. A single recessive gene in ‘Modjo’ barley mediating resistance to the California “E” isolate of BSMV during seedling tests was reported in the 1950s [12], [17] and a single recessive gene also was reported to mediate resistance to BSMV strain ND1 in the barley cultivars ‘Traill’ (CI 9538), ‘Modjo-1’, and ‘Moreval’ (CI 5724) [18], [19]. Subsequently, Timian and Franckowiak [20] identified a single recessive gene designated *rsm1* that confers resistance to BSMV strain CV42 in ‘Modjo-1’, ‘Moreval’, and ‘CI 4197’ barley, and were able to map the gene to the *Lk2* locus (controlling awn length) near the centromere of the long arm of chromosome 7H. Edwards and Steffenson [21] also identified a similar resistance gene to CV42 in ‘Morex’ which was provisionally designated *Rsm1Mx* and was closely linked to the restriction fragment length polymorphism (RFLP) marker ABC455 near the centromere of chromosome 7H. Although it is possible that the genes evaluated in the latter two studies are identical, differences among the virus strains and barley genotypes in the studies prevent definitive identification of their relationships. Unfortunately, the resistance genes are located near the centromere in a region of low recombination rate, which greatly reduces the possibility of using positional cloning approaches to further refine their relationships.

*Brachypodium distachyon* (henceforth Brachypodium) is a member of the *Poaceae* subfamily *Pooideae* and has emerged as a model species for the study of cool season cereal crops (barley, wheat, oats and rye). This small plant is easy to cultivate, has a small genome, a short life cycle, is self-fertile and has a large amount of genetic variation [22]–[26]. During the past decade, a community of researchers has concentrated on generating a large array of resources for molecular genetics and genomics research in Brachypodium [27]. These include recombinant inbred lines, efficient transformation methods, T-DNA insertion lines, BAC libraries, BAC end sequences, ESTs and genetic linkage maps [28]–[39]. A major step forward was the publication of a high quality draft genome sequence for inbred line Bd21 [40]. The availability of these resources makes it possible to efficiently map and clone Brachypodium genes controlling many traits including disease resistance.

Although, analysis of pathogens infecting Brachypodium is in its infancy, infections with the rice blast pathogen [41], crown rust, stem rust and stripe rust [22], and Fusarium head blight [42] have been described. High levels of colinearity between the genome of *Brachypodium sylvaticum* and cool season cereal crops have facilitated identification of both the wheat leaf rust resistance gene *Lr34* [43] and the domestication locus *Q* [44]. We recently found that several BSMV strains are able to infect Brachypodium. Among these, the BSMV ND18 strain is able to infect inbred line Bd21, with infected plants containing a large amount of virus, and exhibiting intense mosaic symptoms, stunting and failure to set seeds. In contrast, inbred line Bd3-1 exhibits a high degree of resistance and does not produce visible mosaic symptoms or contain detectable amounts of virus at 20 to 25°C.

Here we describe the genetic and biological properties of BSMV resistance in Brachypodium inbred line Bd3-1 including fine mapping of a putative resistance gene, designated *Bsr1,* to a five gene locus. As part of this work, we created a F_6∶7_ Brachypodium RIL population and developed a SNP-based genetic linkage map to identify the approximate recombination break points across the genomes of all RILs to create a valuable genetic resource with wide ranging applications. Future cloning and characterization of *Bsr1* should shed light on host factors affecting BSMV virulence in cereals and may provide new resistance resources for barley and wheat breeding programs.

**Table 1:** Disease responses of diverse *Brachypodium distachyon* lines.

Inbred line	Visual Phenotype	Serological Results	Origin
Adi-1	Susceptible	Positive	Turkey
Adi-10	Resistant	Negative	Turkey
Adi-11	Susceptible	Positive	Turkey
Adi-12	Susceptible	Positive	Turkey
Adi-15	Susceptible	Positive	Turkey
Adi-2	Susceptible	Positive	Turkey
Adi-21	Susceptible	Positive	Turkey
Adi-23	Resistant	Negative	Turkey
Adi-3	Resistant	Negative	Turkey
Adi-4	Susceptible	Positive	Turkey
Adi-6	Susceptible	Positive	Turkey
Adi-7	Susceptible	Positive	Turkey
Adi-8	Resistant	Negative	Turkey
Adi-9	Susceptible	Positive	Turkey
Bd21	Susceptible	Positive	Iraq
Bd21-3	Susceptible	Positive	Iraq
Bd2-3	Susceptible	Positive	Iraq
Bd3-1	Resistant	Negative	Iraq
BdTR10C	Susceptible	Positive	Turkey
BdTR11I	Susceptible	Positive	Turkey
BdTR12C	Susceptible	Positive	Turkey
BdTR13C	Susceptible	Positive	Turkey
BdTR2G	Susceptible	Positive	Turkey
BdTR3C	Susceptible	Positive	Turkey
BdTR5I	Susceptible	Positive	Turkey
BdTR9K	Susceptible	Positive	Turkey
Bis-1	Susceptible	Positive	Turkey
Bis-4	Susceptible	Positive	Turkey
Bis-5	Susceptible	Positive	Turkey
Gaz-4	Resistant	Negative	Turkey
Gaz-5	Resistant	Negative	Turkey
Gaz-8	Susceptible	Positive	Turkey
Gaz-9	Susceptible	Positive	Turkey
Kah-2	Susceptible	Positive	Turkey
Kah-4	Susceptible	Positive	Turkey
Kah-5	Susceptible	Positive	Turkey
Koz-3	Susceptible	Positive	Turkey
Koz-4	Resistant	Negative	Turkey
Koz-6	Resistant	Negative	Turkey
Koz-7	Susceptible	Positive	Turkey
Tek-1	Susceptible	Positive	Turkey
Tek-10	Susceptible	Positive	Turkey
Tek-12	Susceptible	Positive	Turkey
Tek-2	Susceptible	Positive	Turkey
Tek-3	Susceptible	Positive	Turkey
Tek-4	Resistant	Negative	Turkey
Tek-5	Resistant	Negative	Turkey
Tek-9	Resistant	Negative	Turkey

## Materials and Methods

### Brachypodium Germplasm and Recombinant Inbred Lines

Forty-eight previously described inbred lines originating from Turkey and Iraq were used in this study [37], [39], [45]. In addition, a Brachypodium RIL population was generated from a cross between inbred lines Bd3-1 (female) and Bd21 (male). A single F_1_ plant was self-pollinated and the resulting F_2_ seeds were propagated by single seed descent to the F_6_ generation. Individual F_6_ plants were then selfed to produce 165 F_6∶7_ RILs for use in genetic analysis and gene mapping. To determine the genetic inheritance pattern of *Bsr1*, a second population consisting of 57 F_2_ plants was generated from a separate Bd21 × Bd3-1 cross.

### BSMV Maintenance and Infectivity Analyses

The BSMV ND18 strain used throughout the study was maintained in a greenhouse by mechanical transfers every 10 to 14 days to the “Black Hulless” barley (*Hordeum vulgare* L.) cultivar at the two-leaf stage [46]. Inoculum for mechanical transmission of Brachypodium plants was produced from infected barley leaves ground in 10 mM sodium phosphate buffer (pH = 7.0) containing 0.5% sodium sulfite and 1% Celite, to produce a leaf extract. Mechanical inoculation was performed by gently rubbing *Nicotiana* leaves with the inoculum, or in the case of barley and Brachypodium by holding the base of the plant with one hand while gently traversing wetted fingers of the other hand from the base to the tip of the leaf to produce a faint squeaking sound. In all cases, care was taken not to injure leaves during rubbing, and after inoculation plants were misted with a gentle spray of water and maintained under shaded conditions to prevent wilting.

To facilitate uniform germination and growth of the Brachypodium plants, seeds were placed in petri plates with damp filter paper in darkness at 4°C for two weeks, then planted in 9 cm square plastic pots containing a sandy-loam soil filled to 2 cm from the top of the pot and covered with 1 cm of fine silica sand. Plants were grown under greenhouse conditions with care taken to prevent temperatures from exceeding 25°C.

In the initial survey to evaluate the lines from Turkey and Iraq, plants were inoculated with infected barley leaf sap when the third leaf had fully expanded. The inoculated plants were maintained in the greenhouse at ∼25°C and visual infectivity determinations on leaves emerging above the inoculated leaves were recorded at 7, 10 and 14 days post inoculation (dpi). RIL trials were carried out in a growth cabinet at ∼22°C with a 12 hr photoperiod under a light intensity of 2500 to 3000 lumens. Infectivity trials were repeated twice with five seeds per genotype and Bd3-1 and Bd21 controls were included in each trial. The presence of BSMV coat protein in leaves of each plant was evaluated at 10 dpi by Enzyme Linked Immunosorbant Assays (ELISA) with a polyclonal antibody raised against purified virus preparations. Reverse transcription polymerase chain reactions (RT-PCR) to assess accumulation of viral RNAs in leaves were carried out as previously described [46], [47].

Infection responses (IR) on a scale of 1 to 4 were determined on leaves emerging above the inoculated leaves by using a combination of visual symptoms and serological reactions. Plants given an IR score of 1 failed to develop visible symptoms and were similar in appearance to uninoculated plants, an IR score of 2 represented highly resistant plants with mild necrotic streaks on leaves, and an IR score of 3 was assigned to plants with more extensive necrosis and some wilting, but no mosaic symptoms. In all three cases, plants had negative ELISA responses (<0.05 A_490_) and hence were classified as resistant. Plants given an IR score of 4 developed mosaic symptoms, with or without associated necrosis, and had positive ELISA responses (>0.75 A_490_). Because some resistant and susceptible controls occasionally developed variable amounts of tissue necrosis after inoculation, we could not determine whether the sporadic necrosis was due to virus infection, mild environmental stresses or other microbial infections. Therefore, we used the mosaic symptoms and ELISA results as the primary criteria for classifying infections as susceptible or resistant (Table 1).

### DNA Extraction and Development of SNP, Indel and CAPS Markers

Leaves from individual F_6_ plants selfed to generate RILs were harvested, cut into 2 cm lengths, and 4 sections from each plant were placed in 2 ml polycarbonate tubes specifically designed for multiple sample processing in a 2010 model Geno/Grinder (BT&C Inc., Lebanon NJ) and the tissue samples were frozen and lyophilized. Samples were stored at −80°C prior to being pulverized to a fine powder by adding 3 glass beads (4 mm diameter) to each tube followed by shaking at 1,000 strokes/min for 1 min. Then, 500 µl of hot (65°C) DNA extraction buffer (0.1 M Tris-HCl pH 7.5, 0.05 M EDTA pH 8.0, 1.25% SDS) was added to each tube followed by vigorous shaking and incubation at 65°C for 30 min. Next 250 µl of cold (4°C) 6 M ammonium acetate was added, mixed thoroughly and incubated for 15 minutes on ice before centrifuging for 5 minutes at 16,000 g in a microfuge. The supernatant (600 µl) was transferred to a 1.5 ml microfuge tube, mixed thoroughly with 360 µl of isopropanol, and incubated for 10 to 30 min on ice for DNA precipitation. DNA was pelleted by centrifuging for 10 min at 16,000 g, the supernatant was decanted and tubes were inverted to eliminate residual supernatant, and the pellet was thoroughly washed with 1 ml of 70% EtOH. After drying, the pellet was resuspended overnight in double distilled H_2_O at 4°C, centrifuged briefly to remove undissolved material and stored at −20°C until use.

Single nucleotide polymorphism (SNP) markers from a Bd3-1 × Bd21 F_2_ genetic linkage map [33] were used to genotype the RIL population using the Illumina Golden_Gate assay [48] at the UC-Davis Genome Center. Allele calling for each SNP locus was carried out with GenomeStudio software (Illumina, San Diego, CA) as described by Huo *et al*. [33]. All genotypic data was manually reviewed and re-scored if errors in calling the homozygous or heterozygous clusters were evident. To identify markers closer to the BSMV resistance locus, we examined whole genome resequencing data produced by the DOE Joint Genome Institute. Insertion and deletions (Indels) and single nucleotide polymorphisms (SNPs) around the BSMV resistance locus were used to design Indel markers and cleaved amplified polymorphic sequences (CAPs) markers for fine mapping of the *Bsr1* locus.

**Figure 1:**
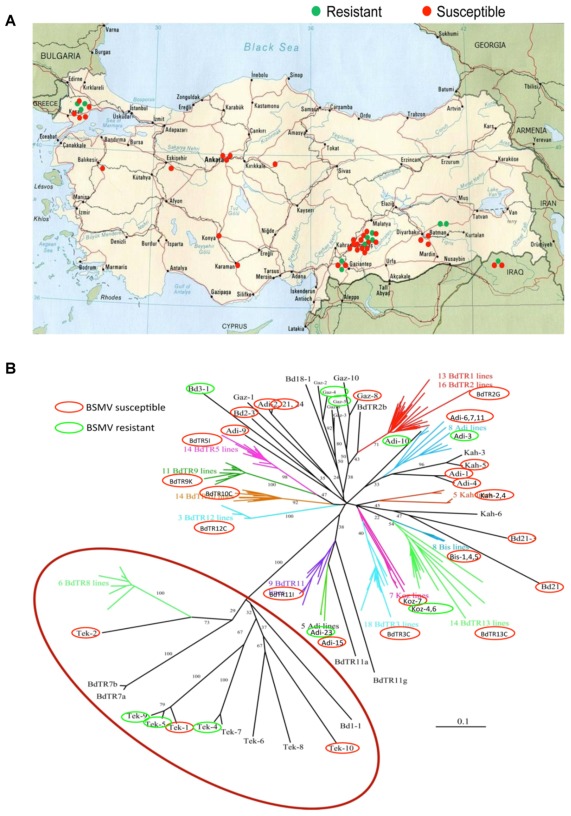
Geographic and genotypic distributions of the phenotype elicited during BSMV ND18 infection of diverse Brachypodium lines. **A**) Infection phenotype of 44 Brachypodium lines from 11 locations in Turkey. Red dots represent the locations of the 33 susceptible lines; Green dots show the distribution of the 11 resistant lines. **B**) Plot of the phenotypic responses of the Brachypodium lines on a previously created neighbor joining tree based 44 SSR markers (Vogel et. al. 2009). Red ovals represent susceptible lines and green ovals show resistant lines.

### Genetic Map Construction

For each polymorphic marker, a χ^2^ analysis was performed and markers that deviated from the expected 1∶1 segregation ratio were discarded. Linkage analysis between markers, estimation of recombination frequencies and determination of the linear order of loci, were performed using JoinMap 4.0 software program using the maximum likelihood mapping algorithm and an initial logarithm of odds score of 10 as described in Ref. [33]. Recombination rates were converted to genetic distances in cM using the Kosambi mapping function.

## Results

### BSMV Infection Responses in Diverse Brachypodium Lines

A screen was carried out to evaluate the resistance or susceptibility of 44 inbred Brachypodium lines from Turkey and 4 lines from Iraq. The lines were selected to be genetically diverse based on simple sequence repeat (SSR) marker analysis [39]. Visual mosaic symptoms were evaluated at 7, 10 and 14 dpi, and ELISA readings were conducted at 10 dpi to quantify the level of virus coat protein. Among these lines, 36 developed mosaic symptoms and reacted positively in ELISA tests and were considered to be susceptible, whereas 12 failed to develop visible infections, had negative ELISA reactions and were designated resistant (Table 1). To determine if resistance correlated with geographic origin, the infection responses of each line from Turkey was superimposed on a map of the collection locations (Figure 1A). To determine possible genetic relationships between resistant lines, the resistant and susceptible lines were highlighted on a previously constructed tree based on SSR markers (Figure 1B). No substantial correlation between BSMV resistance and geographic distribution or genetic relationship was observed.

**Figure 2:**
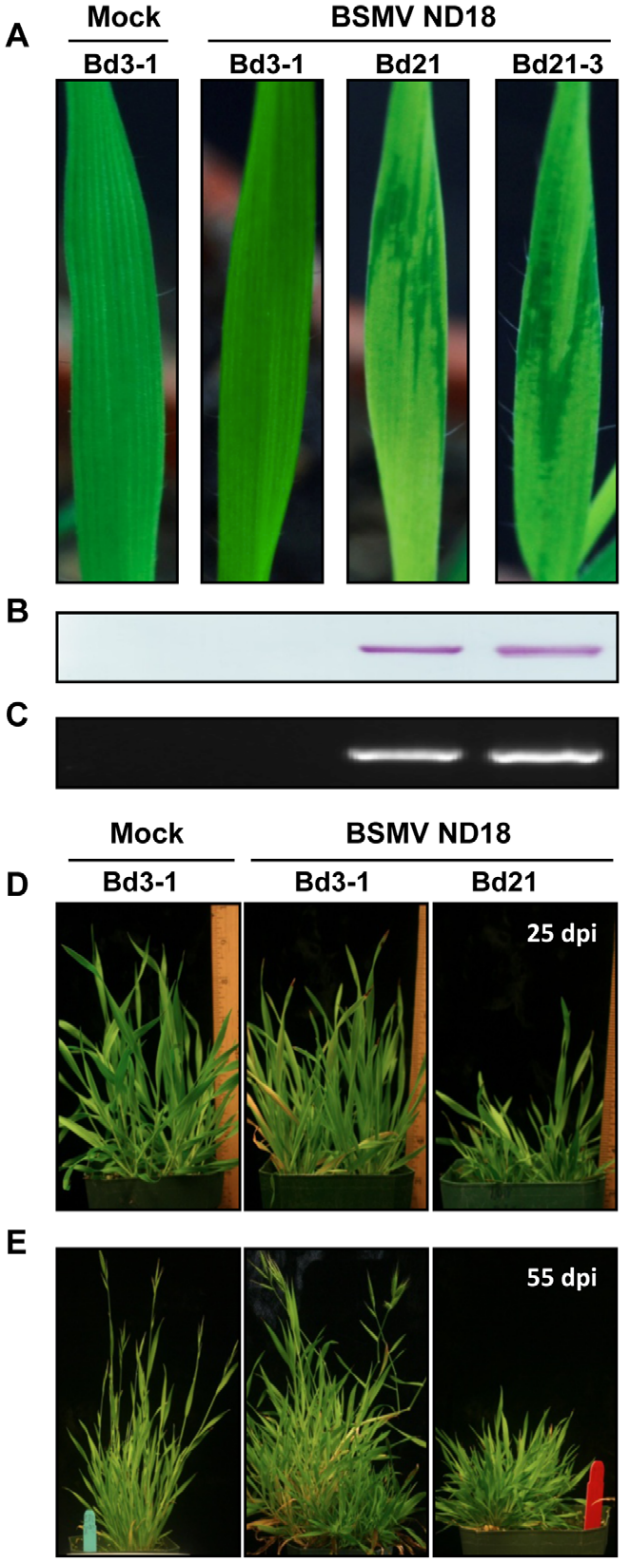
Disease responses of Brachypodium lines Bd3-1, Bd21 and Bd21-3 to infection with BSMV ND18. (**A–C**) Uninfected Bd3-1 and inoculated Bd-3-1, Bd21 and Bd21-3 at 12 dpi. (A) Uninfected Bd3-1 plants remained green and continued to grow rapidly, as was typical of uninfected Bd21 and Bd21-3 plants. Nd18 inoculated Bd3-1 plants failed to develop symptoms and had the same general appearance as their uninoculated counterparts. In contrast, Bd21 and Bd21-3 inoculated plants developed visible mosaic symptoms on emerging leaves by 7 days post inoculation (dpi) and the symptoms remain visible until at least 20 dpi. (**B**) Western blots to determine the presence of the 22 KD BSMV coat protein in leaf extracts from the first emerging leaf of uninoculated and inoculated plants at 6 dpi. (**C**) RT-PCR analyses of leaf extracts taken at 21 dpi from the lines shown in the top panel. A forward primer complementary to the 3′ end of BSMV RNAs and a reverse primer of the same polarity as the γb gene were designed to produce an ∼800 nt product. (**D–E**) Chronic disease symptoms on Brachypodium lines inoculated with BSMV ND18. (**D**) Bd3-1 and Bd21 at 25 dpi. Note stunting of Bd21 compared to Bd3-1. (**E**) Healthy Bd3-1 and Bd 3-1 and Bd21 at 55 dpi. **Note:** Uninoculated plants and inoculated Bd3-1 plants have a similar growth characteristics and seed population, but Bd21 plants are stunted and fail to flower or set seeds.

**Table 2:** Segregation ratios of Bd21 × Bd3-1 F_2_ and Bd3-1 × Bd21F_6∶7_ RIL populations for BSMV ND18 resistance.

Population	Size	Segregation					
		Type	Expected ratio	R	H	S	χ^2^
RILs	165	Single locus	A:B = 1∶1	76	3	86	0.62
F_2_	57	Dominant	(A+H):B = 3∶1	41		16	0.28

**Table 3:** Comparison of SNP-based genetic linkage maps of Bd3-1 × Bd21 F_2_ and F_6∶7_ RIL populations.

	Number of markers		Genetic map length (cM)		cM/marker		Physical size (Mb)	kb/marker		Recombination rate (cM/Mb)		
Type	F_2_	RILs	F_2_	RILs	F_2_	RILs		F_2_	RILs	F_2_	RILs	
Chr 1	152	159	449.1	376.3	3.0	2.4	74.8	492.1	470.4	6.0	5.0	
Chr 2	91	96	348.3	388.0	3.8	4.0	59.3	651.6	617.7	5.9	6.5	
Chr 3	137	136	350.9	418.6	2.6	3.1	59.9	437.2	440.4	5.9	7.0	
Chr 4	112	110	267.0	304.7	2.4	2.8	48.6	433.9	441.8	5.5	6.3	
Chr 5	66	69	182.7	198.0	2.8	2.9	28.4	430.3	411.6	6.4	7.0	
Total	558	570	1598.0	1685.6	2.9	3.0	271.0	485.7	475.4	5.9	6.2	

Note: Chr 1 includes small groups A, B, D; Chr 2 includes small group C.

**Table 4:** Primer sequence and physical location of Indel and CAPS markers used for fine structure mapping of the Brachypodium Bd3-1 × Bd21 F_6∶7_ RIL population.

Marker	Physical locationin Bd21		Correspondinggene in Bd21	Primer sequence		Comments
	Start	End		Left (forward)	Right (reverse)	
Bd3-2	181391	181574	Bradi3g00450	GGTCCAAGAAGCCAATTTCA	ACAACCTCAAGGTGCTCGAC	181484 -10:TAGCGGTAAG
Indel-1	297714	297916		CGAGGACATGGAGCACTTTT	GGCCGAAATTAGGTCTCCTC	297805+11:CTTTCCTATTC
Indel-6	328743	328916	Bradi3g00620	GCCACTAGATCGCCATGACT	TGCATGTGCAACATGTGACT	328831+9:TGAATATGC
Caps-4	371691	372044		GCATCTGGCCTCGCTACTAC	TGCCGGAATAAAACTCCAAG	371773+6:GAGTCG
Caps-5	402366	402756	Bradi3g00730	GCCCTCGATTGCATCTATCT	CGCTACCTGAACCACACAAA	402688 T/C
Caps-9	407773	408091		CGGTGGTCCAGTTCATTTCT	GGAGATGGATGTCCCAGCTA	408018 G/A
Caps-11	419695	419958	Bradi3g00757	AATAAAACCTTCGGCCATCC	GGTTTGCCTCTTGCAATCAT	419812 G/A
Caps-12	424681	425029	Bradi3g00767	GTGTCATCATGGCAATCGAG	ACTGGTTCGTGGAGGTCTCA	424950 G/C
Caps-8	426497	426965	Bradi3g00767	TCCCAGGTCAAGAAGGAGAA	CTTAAGACATGTGCGCTGGA	426590+6:CGCGGG
Indel-4	441377	441529		AAAGTTGCCCCCTTGATTTT	AAGCCACAGAGGAAAGTGGTT	441530 -14:TATTTAGCAGATAC
Indel-2	460601	460839		GCACACGGAACAAGCTAGAAA	GCTCGTGGCTTGTTTGCTAT	460661 -20:ATATAAGTGGTAATGTTGAC
Caps-2	487758	487991		TCTCTGGGCTCTGGCTACAT	CGATAGGCCAGCTCTTCAAC	487847 A/G
Caps-3	525604	525912	Bradi3g00940	TCTCCTCCAGGCAGATTGTT	AGACTGGCAGCCTCACTGAT	525675 T/C
Bd3-6	607733	607982		TGGAGATGGGCTTTAGGAGTT	GCTGGAAAACATTTTGGAGAA	607813 -23:CTACCCATATCACTTGTCTCGAA

**Figure 3:**
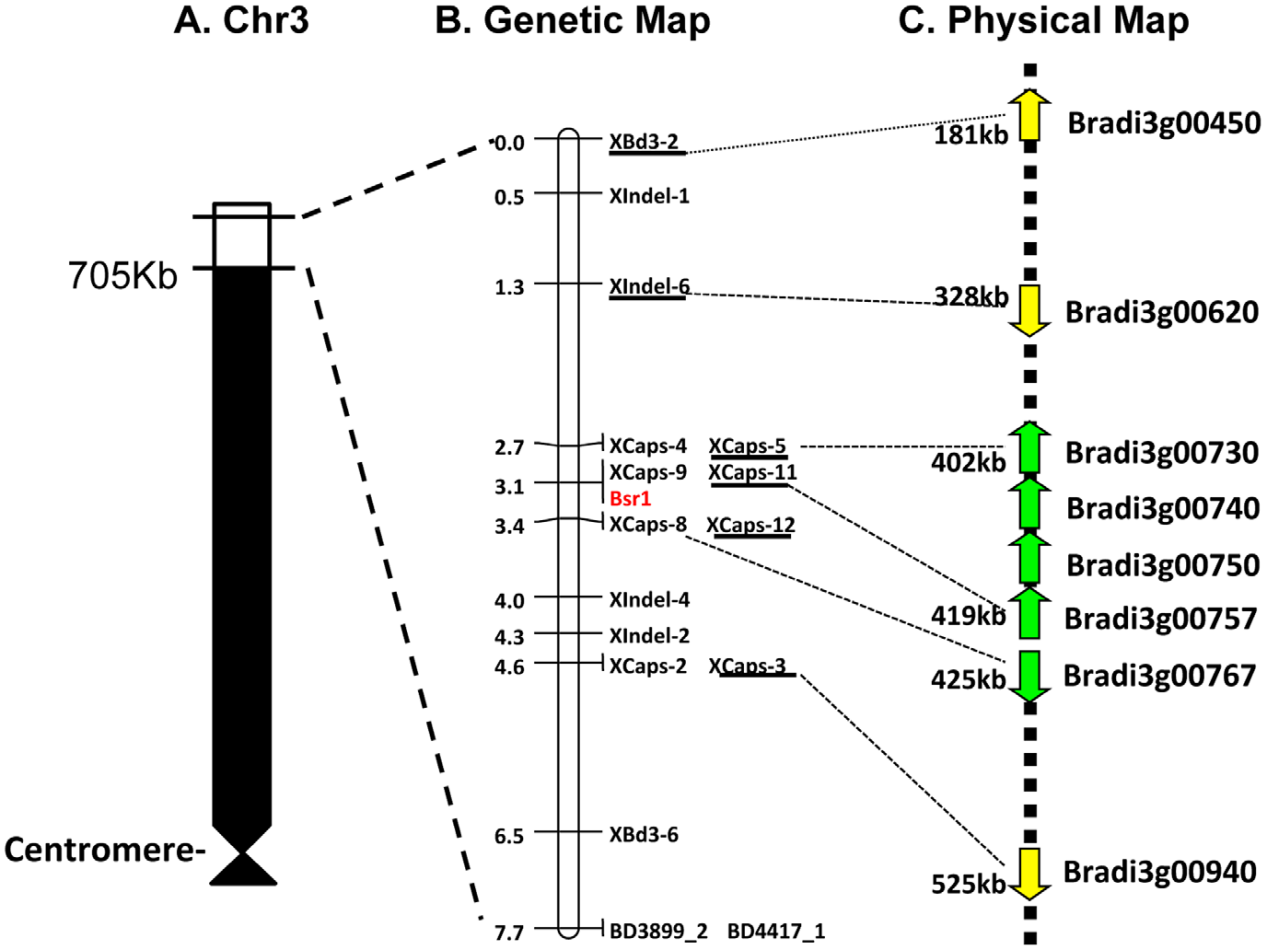
Genetic map of BSMV *Bsr1* resistance within the distal region of Bd21 chromosome 3. **(a)** **Cartoon of the short arm of Bd21 chromosome 3 (Chr3S).** The white region shows 705 kb of distal region of Chr 3 encompassing the fine mapping region. **(b)** **Genetic map of the 705** **Kb region of chromosome 3.** Markers are shown on the right with map distances on the left. The furthest flanking markers that were previously assigned to the Brachypodium Chr 3 are indicated by dashed lines. The *Bsr1* locus is indicated in red. The six markers located within the predicted Bd21 locus served as anchors to establish co-linearity between the *Bsr1* genetic map and the physical map of Bd21. **(c) Physical map of the** ***Bsr1*** **interval.** Annotated genes are indicated by arrows and candidate genes are indicated in green. Approximate locations (bp) are shown on the left.

Brachypodium inbred lines Bd3-1, Bd21 and Bd21-3, all from Iraq, are of particular interest because of the resources available for these lines. The phenotypic responses of Bd3-1, Bd21 and Bd21-3 on emerging leaves inoculated with ND18 along with western blot analyses of coat protein, and RT-PCR of sequences common to the 3′ sequences of the three genomic RNAs of the virus are shown in Figure 2A–C. These experiments revealed that Bd21 and Bd21-3 are susceptible to ND18 because both inbred lines developed mosaic symptoms within 7 dpi and accumulated high levels of virus RNA and protein, whereas Bd3-1 failed to develop symptoms and did not contain detectable viral RNA or protein. After 3 weeks, substantial stunting of infected plants was evident and the plants failed to set seeds upon maturity (Figure 2D and 2E). In contrast, Bd3-1 failed to develop mosaic symptoms and its growth and seed production was indistinguishable from uninoculated plants.

### Temperature Sensitivity of Bd3-1 Resistance

In our initial greenhouse evaluations of phenotypic responses to BSMV infection, we noted that as the temperature and light intensity increased during the early summer, resistance began to break in some inoculated Bd3-1 plants and mild mosaic symptoms accompanied by positive ELISA reactions began to appear. To obtain additional detail about the temperature effects on BSMV resistance, Bd3-1 and Bd21 plants were grown at 21 to 24°C in a growth chamber and transferred to 20, 25, 27 and 30°C growth chambers with lighting conditions similar to those used for the RIL screening assays. The results revealed that resistance was maintained up to 25°C, but at or above 27°C some plants began to develop mosaic symptoms and were positive for the presence of BSMV CP and RNA (data not shown).

### Genetic Analysis of BSMV Resistance in Bd3-1

The inheritance of BSMV resistance in Bd3-1 was determined by evaluating the resistance or susceptibility to ND18 of F_2_ plants resulting from a cross between Bd3-1 and Bd21. The 57 F_2_ plants segregated as 41 resistant and 16 susceptible, which fits a 3∶1 Mendelian ratio expected for a single dominant gene (Table 2; See Table S1). Additional infectivity results with plants comprising the 165 F_6∶7_ RILs revealed a segregation ratio of 76 resistant and 86 susceptible lines, with 3 RILs still segregating. This segregation pattern fits the expected 1∶1 ratio for a single gene and indicates that a single locus in Bd3-1 is responsible for resistance to ND18. These results provide strong evidence that a single dominant gene confers resistance to BSMV ND18 in Bd3-1, and we designated the gene *Barley stripe resistance 1* (*Bsr1*).

### Rough Mapping of *Bsr1* and Identification of Recombination Breakpoints in RIL Population

To define the approximate recombination break points in the RILs, we genotyped the RIL population using a set of 768 previously identified SNP markers [33]. Of these markers, 198 failed to produce high-quality genotype data and could not be mapped. This result is similar to that obtained with 476 F_2_ individuals from the same cross. In this case, 200 markers failed to produce useable genotype data [33]. Significantly, in both cases all markers that produced good genotype data were mapped to a unique position. In the present study, 570 markers, including 34 pairs of control markers that were within 500 kb of one another, and BSMV resistance phenotypic data were used to construct a linkage map for the F_6∶7_ population using the JoinMap4 program (Figure S1). Five major linkage groups containing 550 markers were identified. These correspond to the five chromosomes of Brachypodium and their genetic lengths ranged from 198 cM for chromosome 5 (Chr 5) to 418.6 cM for Chr 3, with an aggregate length of 1685.6 cM (Table 3, Table S2). In addition to the five major linkage groups, four small linkage groups A, B, C and D, consisting of 6, 6, 4 and 4 SNP markers, respectively, were identified (Figure S1). These four small linkage groups were linked to chromosomal linkage groups (group A and D on the top of Chr 1, group B on the bottom of Chr 1, and group C on the top of Chr 2) in the F_2_ map, suggesting that the additional recombination and smaller size of the RIL population used here led to our failure to observe linkage to a chromosome. Mapping of *Bsr1* on the RIL SNP map indicated that it is located 5.6 cM above marker BD3899 at the top of Chr 3, and within 705 kb of the distal end of the short arm of Chr 3. In addition, we determined the approximate recombination breakpoints for each individual RIL. For this exercise, we assigned the four small linkage groups to chromosomal locations based on previous F_2_ mapping data and the genome assembly [33], [40] (See Figure S1; Table S3).

### Fine Mapping of *Bsr1*

To refine the map position of *Bsr1*, six Indel markers and eight CAPS makers were developed based on whole genome resequencing data from the DOE Joint Genome Institute (Table 4). These markers were used to create a new genetic linkage map for the distal 700 kb of chromosome 3 and indicated that *Bsr1* lies in a 0.7 cM interval between the XCAPS-5 and XCAPS-12 markers and co-segregates with the XCAPS-9 and XCAPs-11 markers (Figure 3). The genetic order of the markers in this region was consistent with their physical locations on Chr 3, indicating that the genome assembly is correct in this region. Thus, *Bsr1* lies within an ∼23 kb genomic region that contains five gene models, Bradi3g00730, Bradi3g00740, Bradi3g00750, Bradi3g00757 and Bradi3g00767. BLAST and protein domain analysis of these gene models revealed that Bradi3g00730 is similar to a MADS-box transcription factor containing a SRF-TF domain; Bradi3g00757 has homology to a resistance gene whose putative product contains an NB-ARC domain and an LRR domain; Bradi3g00767 is related to an antifreeze protein; and Bradi3g00740 and Bradi3g00750, have no predicted function.

## Discussion

Analysis of the BSMV ND18 infection phenotype on a diverse collection of Brachypodium lines indicates that BSMV resistance is not correlated with geographic distribution or SSR genotype. Hence, it is likely that BSMV resistance may have arisen multiple times during Brachypodium evolution and that the virus may have exerted strong selection pressure for maintenance of one or more genes that limit virus infection, since infected plants set few seeds. Although our infectivity screen does not indicate whether or not the resistance of different genotypes is solely due to *Bsr1* or distinct genes, several BSMV strains are able to overcome *Bsr1* resistance (unpublished), suggesting co-evolution of host resistance and pathogen virulence. The principal host of BSMV in cultivated cereals is barley, which originated from wild barley, *Hordeum vulgare* ssp. *spontaneum,* approximately 10,000 years ago in the Fertile Crescent [49]. The northernmost arc of the Fertile Crescent includes portions of the collection area for Brachypodium genotypes surveyed in this study; hence, it is possible that BSMV originated in the Fertile Crescent and is an ancient resident that may have been maintained in populations of wild grasses by mechanical transfer and seed transmission in a manner similar to its modern day survival in barley. Therefore, we anticipate that studies of BSMV resistance originating in Brachypodium may contribute to our understanding of natural evolution of resistance and also may provide novel resistance genes suitable for incorporation into barley and other cereals.

The patterns of inheritance in the Bd21 X Bd3-1 F_2_ and Bd3-1 × Bd21 RIL populations are consistent with *Bsr1* being a single dominant resistance gene. In this regard, *Bsr1* differs from the recessive BSMV resistance genes that have been studied in barley [16]. Although it is generally thought that barley cultivars may harbor five BSMV resistance genes, these have not been clearly defined because of differences in BSMV isolates and barley varietal genotypes. Only two detailed mapping studies of BSMV resistance genes have been reported [20], [21], and both studies indicate that BSMV resistance (designated *rsm1* and *RsmMx*) is conferred by a single gene located near the centromere of barley chromosome 7H. Hence, it is possible that *rsm1* and *RsmMx* are the same gene and that some of the five currently accepted genes may be identical [21]. The recessive nature of the BSMV resistance genes in barley varieties suggests that resistance may be due to the absence of a host function required for virus infectivity. The required function is probably encoded by a multifunctional protein needed for host metabolic or regulatory processes, which has acquired a mutation preventing necessary interactions with a critical virus factor required for replication or movement. The *rsm1* resistance in Modjo-1, Moreval, CI 4197, and Morex barley, which constrains the BSMV CV42 strain, is observed in protoplasts in which virulent BSMV ND18, but not CV42 is able to establish infections. This result suggests that *rsm1* resistance is due to the lack of a host factor required for BSMV replication [50]. However, a different result occurs with an uncharacterized gene in oat that imparts resistance to several BSMV strains [51], [52]. In this case, BSMV strains unable to infect oat plants were able to infect oat protoplasts, suggesting that movement or functions downstream of replication are affected by the oat resistance gene. Interestingly, substitution of a similar sequence segments within the virulent isolate ND18 αa replicase protein were able to overcome both the oat and barley resistances in whole plants. However, different amino acids within the substituted region were required to circumvent the oat and barley resistances, respectively [51], [52]. Thus, *rsm1* in barley appears to target replication processes, whereas oat resistance appears to be conditioned by some factor other than replication, such as local or long distance movement, or an inability to silence suppressors of gene silencing.

Brachypodium is a well-suited host for genetic analysis of BSMV genes because inbred line Bd21 is highly susceptible and Bd3-1 is highly resistant to the ND18 strain. However, care must be taken to maintain stable environmental conditions after inoculation because *Bsr1* resistance is temperature-sensitive. Under the conditions employed in this study, visual symptoms were verifiable by serological assays. In some instances, both Bd3-1 and Bd21 plants developed variable necrotic responses that do not appear to be due to BSMV susceptibility; rather, these responses may be due to other pathogens or environmental stresses. The necrosis did not affect *Bsr1* resistance; hence the final resistance scoring relied primarily on the visual mosaic appearing during the first two weeks of infection and the serological results taken at 10 dpi when virus accumulation in susceptible emerging leaves was anticipated to reach a high titer.

The genetic map created from the F_6∶7_ RILs and the genetic map previously created from 476 F_2_ individuals [33] were nearly identical. Indeed, out of the 570 markers contained in the F_6∶7_ RIL map, the positions of only three disagreed with their physical locations. The discrepancy between the genetic and physical locations of one marker, BD5097_1 on Chr 1, was supported by the genotypes of 10 RILs and thus may indicate an area where the physical assembly is incorrect. By contrast, the discrepancy between the genetic and physical locations of the other two markers (BD2239_1 on Chr 3 and BD3052_2 on Chr 5) were only supported by one RIL genotype for each marker, so we cannot be certain that in these two cases the physical order is incorrect. The high degree of marker order conservation indicates that both maps are correct. The presence of several small linkage groups in the F_6∶7_ map is likely due to the greater number of opportunities for recombination and a smaller population size. These small linkage groups could be aligned to the ends of Chr 1 and Chr 2 based on the genome sequence and the F2 map (Figure S1; Table S3). The difference in recombination rate between the maps, 6.2 cM/Mb for the F_6∶7_ map and 5.9 cM/Mb for the F_2_ map, is quite modest given the differences in both population size and structure. Indeed this difference led to similar overall map lengths: 1685.6 cM for the F_6∶7_ map and 1598 cM of the F_2_ map [33]. Brachypodium has already been noted to have a high recombination rate given its genome size [33] and thus it appears that additional recombination occurred as the genotypes of the RILs approached fixation, which increased map length in the RIL population.

Generation of the genetic linkage map for the RILs permitted rapid mapping of *Bsr1* to the top of Chr 3, and additional markers developed from Bd3-1 resequencing data allowed *Bsr1* to be further localized to a 23 kb region that contains five ORFs. One of these ORFs has NB-ARC and LRR domains common in plant R genes; in Brachypodium there are approximately 180 such genes [40]. While the NBS-LRR gene appears to be a good candidate for *Bsr1*, the involvement of the other four genes within the 23 kb region cannot be ruled out based on putative functional annotation alone. The candidate NBS-LRR gene appears to be similar to a number of dominant resistance genes with NBS-LRR domains cloned from dicot hosts that are known to affect either virus replication or cell-to-cell movement [53]–[56]. In the case of *Bsr1*, reverse genetics studies with BSMV strains have revealed that cell-to-cell movement processes are affected and that mutations in the triple gene block 1 movement gene can overcome resistance (unpublished data).

In addition to increasing our understanding of virus-plant interactions, this study clearly demonstrates the feasibility of Brachypodium for forward genetic studies. The RIL population will prove useful for many traits and is already being used to study disease interactions, drought tolerance and cell wall composition [27]. The identification of the approximate recombination break points will greatly accelerate future mapping studies because users will simply have to combine their phenotypic data with the genotypic data for the RIL population to map their genes or QTLs. Furthermore, the high recombination rates observed in this Brachypodium cross will help minimize the number of individuals necessary to map a gene to a small interval. This was clearly demonstrated by our mapping of *Bsr1* to a 23 kb interval with only 165 RILs.

## Supporting Information

Figure S1 SNP linkage map of Brachypodium distachyon.
(PDF)

Table S1 Infection responses of the Bd3-1 × Bd21 RIL population to BSMV ND18 infection.
(DOC)

Table S2 Mapped SNP markers in the Bd3-1 × Bd21 RIL population.
(DOC)

Table S3 SNP mapping data of the Bd3-1 × Bd21 RILs.
(XLS)
