# Supplementary figures and images for: Fine Mapping of the Bsr1 Barley Stripe Mosaic Virus Resistance Gene in the Model Grass Brachypodium distachyon
Source: PLoS One. 2012 Jun 4;7(6):e38333. doi: 10.1371/journal.pone.0038333 (PMC3366947; doi:10.1371/journal.pone.0038333)

# Chromosome 1

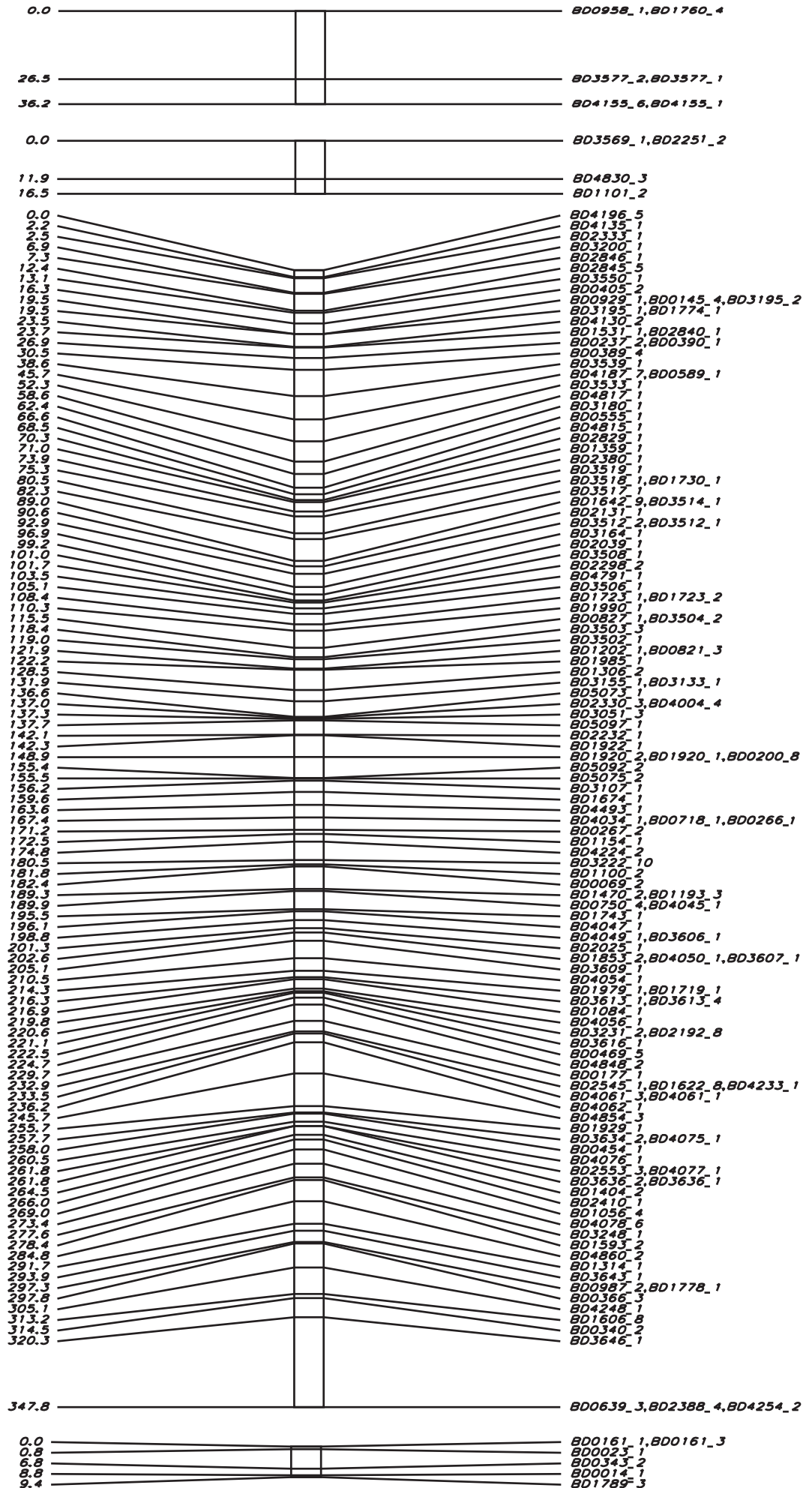

# Chromosome 2

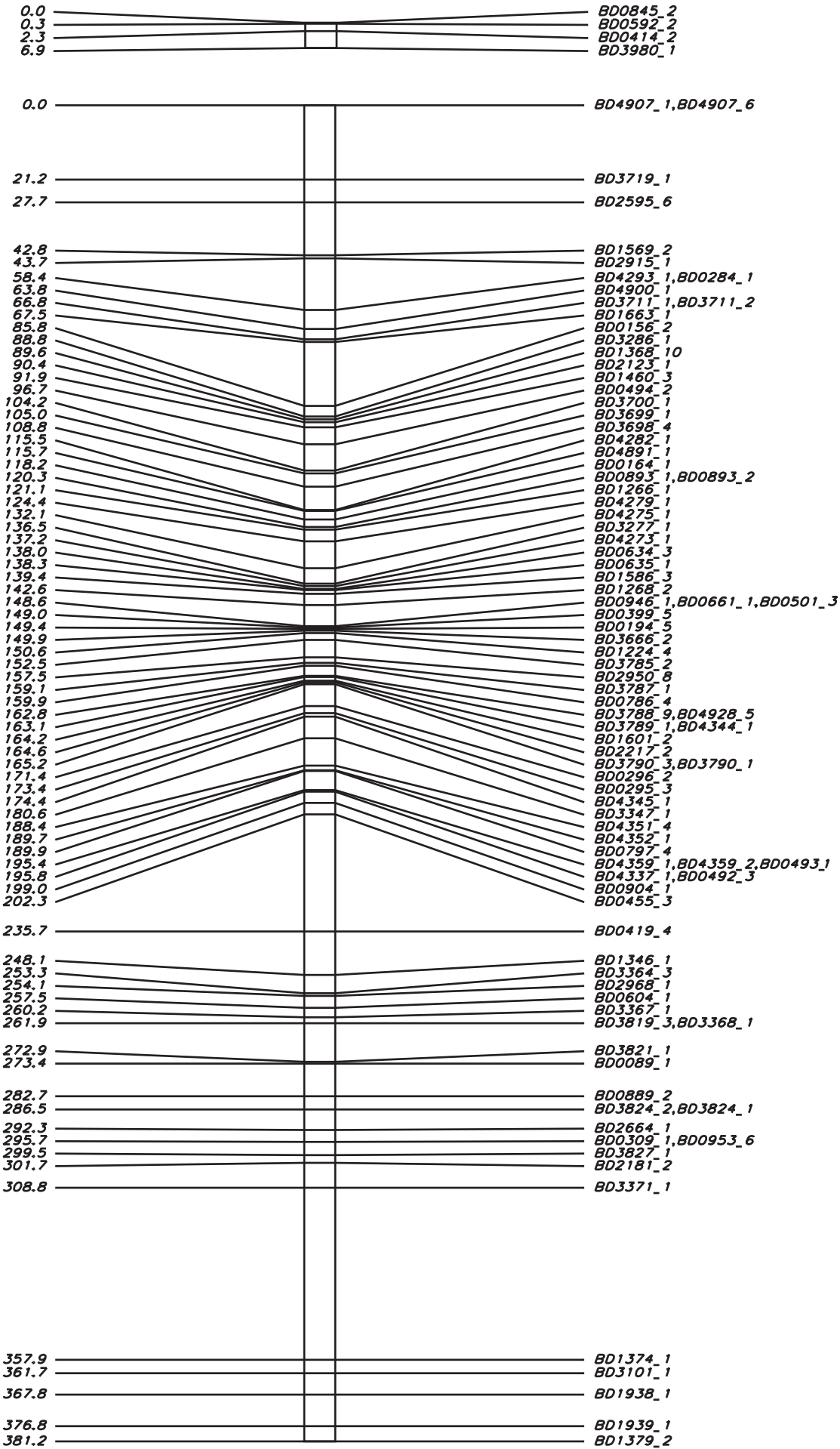

# Chromosome 3

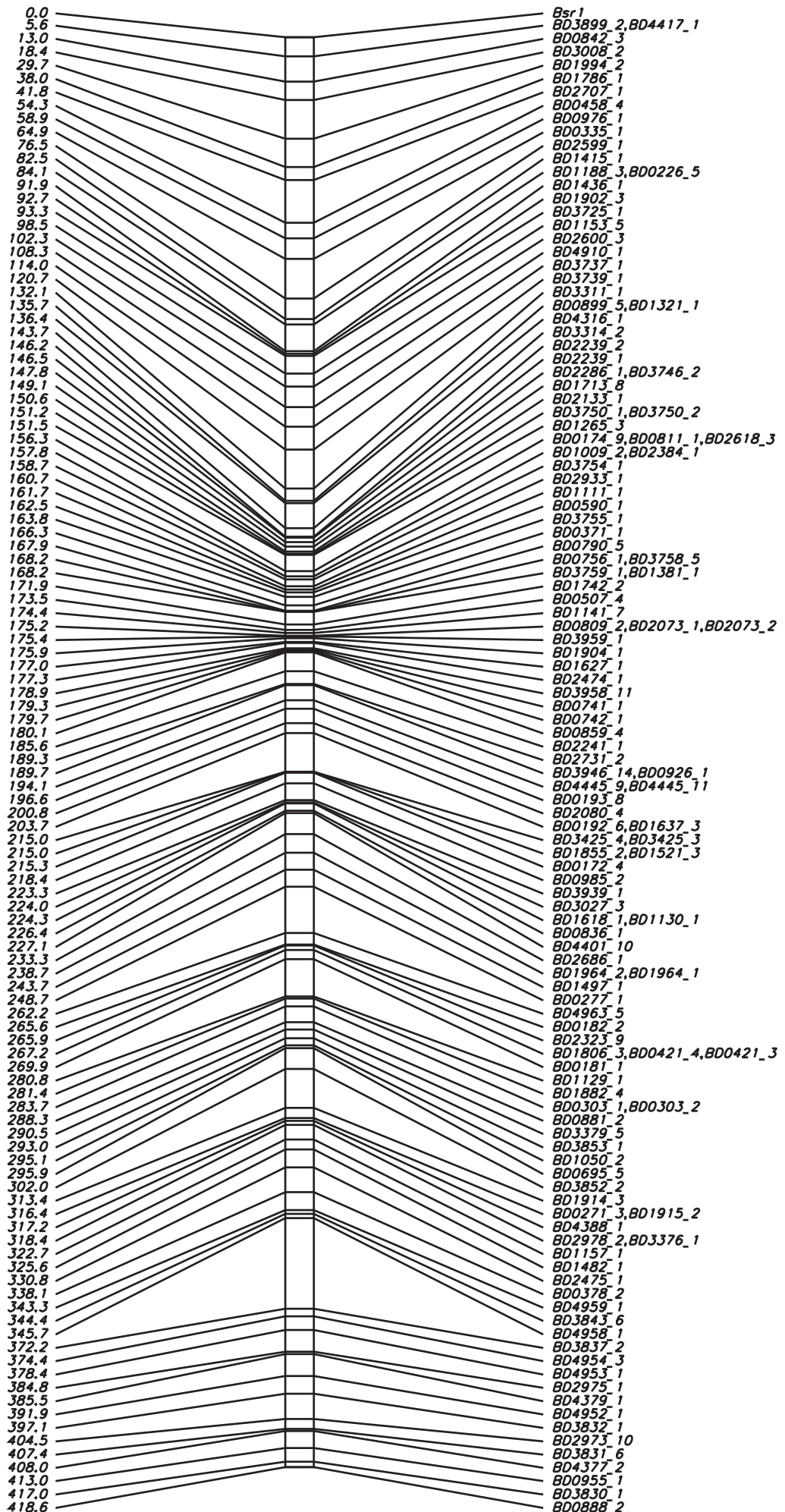

# Chromosome 4

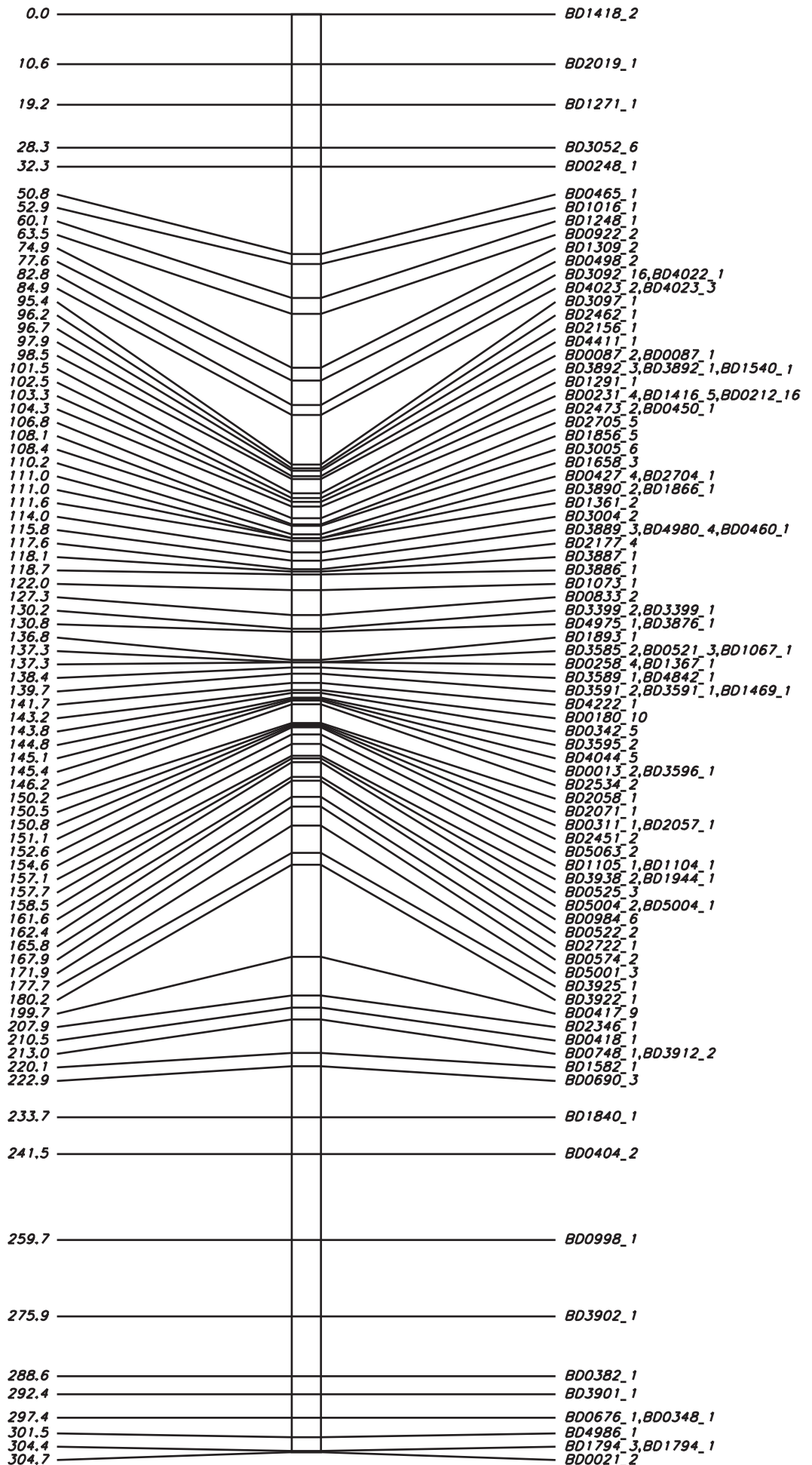

# Chromosome 5

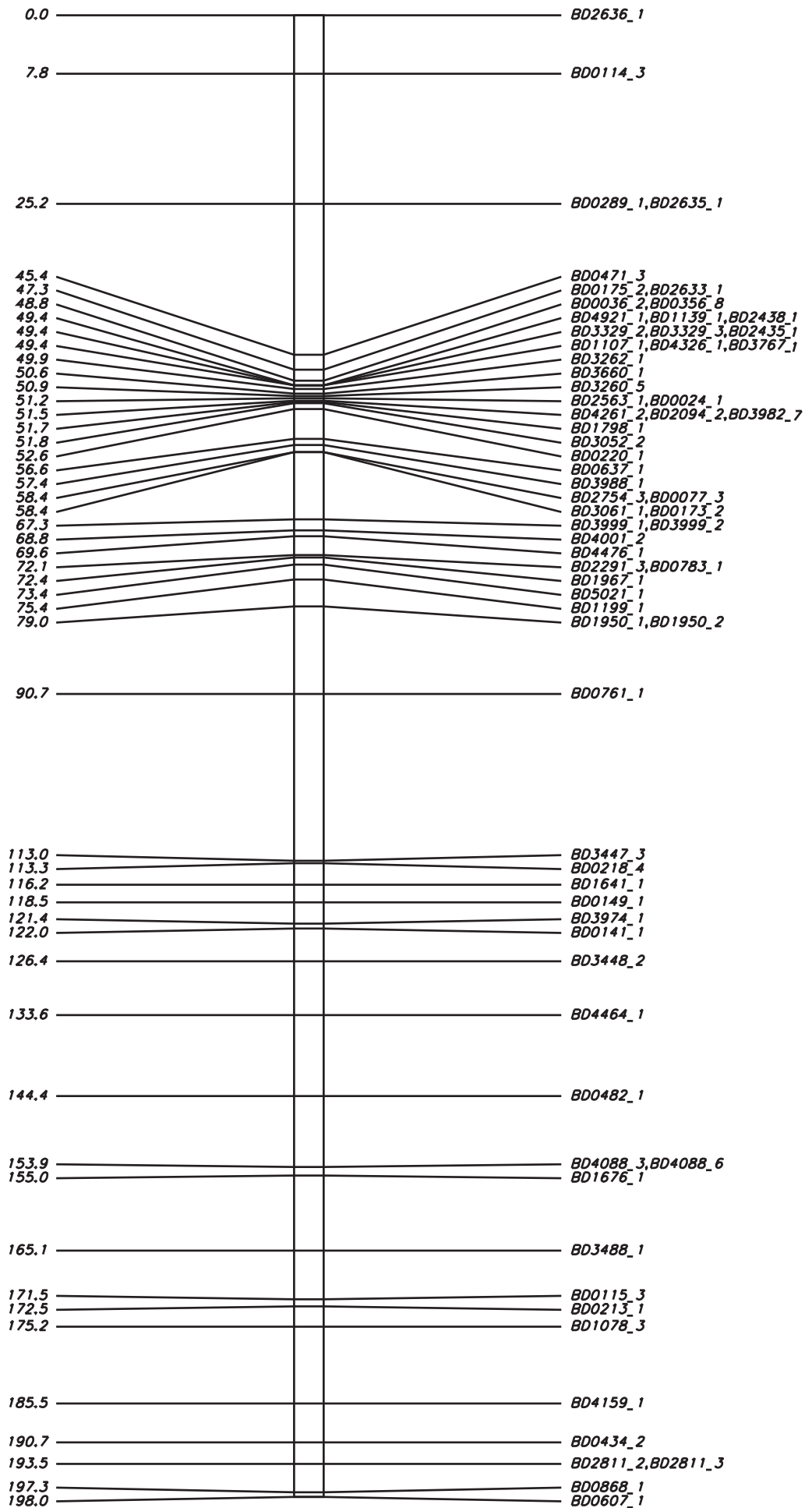

Supplement: Figure S1 — SNP linkage map of Brachypodium distachyon. (PDF) [file pone.0038333.s001.pdf]
