# Supplementary material for: Fine Mapping of the Bsr1 Barley Stripe Mosaic Virus Resistance Gene in the Model Grass Brachypodium distachyon
Source: PLoS One. 2012 Jun 4;7(6):e38333. doi: 10.1371/journal.pone.0038333 (PMC3366947; doi:10.1371/journal.pone.0038333)
Supplement: Table S1 — Infection responses of the Bd3-1 × Bd21 RIL population to BSMV ND18 infection. (DOC) [file pone.0038333.s002.doc]

Table S1. Infection responses of the Bd3-1 X Bd21 RIL population to BSMV ND18 infection

| **RIL #** | **Phenotype** | **Serology** | **RIL #** | **Phenotype** | **Serology** |
| --- | --- | --- | --- | --- | --- |
| Bd21 | Susceptible | 0.75 | RIL009 | Susceptible | Positive |
| Bd3-1 | Resistant | 0.05 | RIL010 | Susceptible | Positive |
| RIL002 | Resistant | Negative | RIL012 | Susceptible | Positive |
| RIL003 | Resistant | Negative | RIL013 | Susceptible | Positive |
| RIL008 | Resistant | Negative | RIL015 | Susceptible | Positive |
| RIL011 | Resistant | Negative | RIL019 | Susceptible | Positive |
| RIL016 | Resistant | Negative | RIL020 | Susceptible | Positive |
| RIL017 | Resistant | Negative | RIL021 | Susceptible | Positive |
| RIL018 | Resistant | Negative | RIL022 | Susceptible | Positive |
| RIL025 | Resistant | Negative | RIL023 | Susceptible | Positive |
| RIL028 | Resistant | Negative | RIL024 | Susceptible | Positive |
| RIL029 | Resistant | Negative | RIL026 | Susceptible | Positive |
| RIL030 | Resistant | Negative | RIL027 | Susceptible | Positive |
| RIL031 | Resistant | Negative | RIL032 | Susceptible | Positive |
| RIL034 | Resistant | Negative | RIL033 | Susceptible | Positive |
| RIL035 | Resistant | Negative | RIL037 | Susceptible | Positive |
| RIL036 | Resistant | Negative | RIL038 | Susceptible | Positive |
| RIL047 | Resistant | Negative | RIL041 | Susceptible | Positive |
| RIL049 | Resistant | Negative | RIL042 | Susceptible | Positive |
| RIL050 | Resistant | Negative | RIL043 | Susceptible | Positive |
| RIL051 | Resistant | Negative | RIL044 | Susceptible | Positive |
| RIL054 | Resistant | Negative | RIL045 | Susceptible | Positive |
| RIL060 | Resistant | Negative | RIL048 | Susceptible | Positive |
| RIL063 | Resistant | Negative | RIL052 | Susceptible | Positive |
| RIL065 | Resistant | Negative | RIL053 | Susceptible | Positive |
| RIL066 | Resistant | Negative | RIL055 | Susceptible | Positive |
| RIL068 | Resistant | Negative | RIL056 | Susceptible | Positive |
| RIL069 | Resistant | Negative | RIL058 | Susceptible | Positive |
| RIL071 | Resistant | Negative | RIL059 | Susceptible | Positive |
| RIL073 | Resistant | Negative | RIL061 | Susceptible | Positive |
| RIL074 | Resistant | Negative | RIL062 | Susceptible | Positive |
| RIL076 | Resistant | Negative | RIL064 | Susceptible | Positive |
| RIL080 | Resistant | Negative | RIL070 | Susceptible | Positive |
| RIL081 | Resistant | Negative | RIL072 | Susceptible | Positive |
| RIL084 | Resistant | Negative | RIL077 | Susceptible | Positive |
| RIL085 | Resistant | Negative | RIL078 | Susceptible | Positive |
| RIL086 | Resistant | Negative | RIL079 | Susceptible | Positive |
| RIL088 | Resistant | Negative | RIL082 | Susceptible | Positive |
| RIL089 | Resistant | Negative | RIL083 | Susceptible | Positive |
| RIL090 | Resistant | Negative | RIL087 | Susceptible | Positive |
| RIL091 | Resistant | Negative | RIL094 | Susceptible | Positive |
| RIL092 | Resistant | Negative | RIL095 | Susceptible | Positive |
| RIL098 | Resistant | Negative | RIL096 | Susceptible | Positive |
| RIL100 | Resistant | Negative | RIL097 | Susceptible | Positive |
| **RIL #** | **Phenotype** | **Serology** | **RIL #** | **Phenotype** | **Serology** |
| RIL102 | Resistant | Negative | RIL099 | Susceptible | Positive |
| RIL103 | Resistant | Negative | RIL101 | Susceptible | Positive |
| RIL106 | Resistant | Negative | RIL104 | Susceptible | Positive |
| RIL107 | Resistant | Negative | RIL108 | Susceptible | Positive |
| RIL111 | Resistant | Negative | RIL109 | Susceptible | Positive |
| RIL115 | Resistant | Negative | RIL112 | Susceptible | Positive |
| RIL119 | Resistant | Negative | RIL113 | Susceptible | Positive |
| RIL121 | Resistant | Negative | RIL114 | Susceptible | Positive |
| RIL123 | Resistant | Negative | RIL116 | Susceptible | Positive |
| RIL126 | Resistant | Negative | RIL117 | Susceptible | Positive |
| RIL127 | Resistant | Negative | RIL118 | Susceptible | Positive |
| RIL128 | Resistant | Negative | RIL120 | Susceptible | Positive |
| RIL129 | Resistant | Negative | RIL122 | Susceptible | Positive |
| RIL130 | Resistant | Negative | RIL124 | Susceptible | Positive |
| RIL131 | Resistant | Negative | RIL125 | Susceptible | Positive |
| RIL136 | Resistant | Negative | RIL132 | Susceptible | Positive |
| RIL137 | Resistant | Negative | RIL135 | Susceptible | Positive |
| RIL142 | Resistant | Negative | RIL138 | Susceptible | Positive |
| RIL143 | Resistant | Negative | RIL139 | Susceptible | Positive |
| RIL146 | Resistant | Negative | RIL140 | Susceptible | Positive |
| RIL147 | Resistant | Negative | RIL141 | Susceptible | Positive |
| RIL152 | Resistant | Negative | RIL144 | Susceptible | Positive |
| RIL153 | Resistant | Negative | RIL145 | Susceptible | Positive |
| RIL156 | Resistant | Negative | RIL149 | Susceptible | Positive |
| RIL158 | Resistant | Negative | RIL154 | Susceptible | Positive |
| RIL160 | Resistant | Negative | RIL155 | Susceptible | Positive |
| RIL161 | Resistant | Negative | RIL157 | Susceptible | Positive |
| RIL163 | Resistant | Negative | RIL159 | Susceptible | Positive |
| RIL164 | Resistant | Negative | RIL162 | Susceptible | Positive |
| RIL166 | Resistant | Negative | RIL165 | Susceptible | Positive |
| RIL175 | Resistant | Negative | RIL168 | Susceptible | Positive |
| RIL176 | Resistant | Negative | RIL169 | Susceptible | Positive |
| RIL181 | Resistant | Negative | RIL170 | Susceptible | Positive |
| RIL183 | Resistant | Negative | RIL171 | Susceptible | Positive |
| RIL001 | Susceptible | Positive | RIL172 | Susceptible | Positive |
| RIL005 | Susceptible | Positive | RIL173 | Susceptible | Positive |
| RIL006 | Susceptible | Positive | RIL174 | Susceptible | Positive |
| RIL007 | Susceptible | Positive | RIL182 | Susceptible | Positive |
| RIL004 | H (3 R, 5 S) | H (3 < 0.1 A_490_, 5 >0.5 A_490_) | | | |
| RIL057 | H (5 R, 5 S) | H (5 < 0.1 A_490_, 5 >0.5 A_490_) | | | |
| RIL150 | H (7 R, 3 S) | H (7 < 0.1 A_490_, 3 >0.5 A_490_) | | | |

Resistant = No visible mosaic and negative ELISA results falling between 0.05 to 0.1 A_490_.

Susceptible = Obvious mosaic symptoms and positive ELISA results above 0.3 A_490_.

Heterozygosis (H) indicates that plants in the line had variable Susceptible (S) or Resistant (R) responses.

Parenthesis shows the numbers of S or R plants in each line.
