# Supplementary material for: Fine Mapping of the Bsr1 Barley Stripe Mosaic Virus Resistance Gene in the Model Grass Brachypodium distachyon
Source: PLoS One. 2012 Jun 4;7(6):e38333. doi: 10.1371/journal.pone.0038333 (PMC3366947; doi:10.1371/journal.pone.0038333)
Supplement: Table S2 — Mapped SNP markers in the Bd3-1 × Bd21 RIL population. (DOC) [file pone.0038333.s003.doc]

Table S2. Mapped SNP markers in the Bd3-1 X Bd21 RIL population

| Locus | Chromosome | Genetic Position (cM) |
| --- | --- | --- |
| BD0958_1 | A | 0 |
| BD1760_4 | A | 0 |
| BD3577_2 | A | 26.5 |
| BD3577_1 | A | 26.5 |
| BD4155_6 | A | 36.2 |
| BD4155_1 | A | 36.2 |
| BD0161_1 | B | 0 |
| BD0161_3 | B | 0 |
| BD0023_1 | B | 0.8 |
| BD0343_2 | B | 6.8 |
| BD0014_1 | B | 8.8 |
| BD1789_3 | B | 9.4 |
| BD0845_2 | C | 0 |
| BD0592_2 | C | 0.3 |
| BD0414_2 | C | 2.3 |
| BD3980_1 | C | 6.9 |
| BD3569_1 | D | 0 |
| BD2257_2 | D | 0 |
| BD4830_3 | D | 11.9 |
| BD1101_2 | D | 16.5 |
| BD4196_5 | Chr1 | 0 |
| BD4135_1 | Chr1 | 2.2 |
| BD2333_1 | Chr1 | 2.5 |
| BD3200_1 | Chr1 | 6.9 |
| BD2846_1 | Chr1 | 7.3 |
| BD2845_5 | Chr1 | 12.4 |
| BD3550_1 | Chr1 | 13.1 |
| BD0405_2 | Chr1 | 16.3 |
| BD0929_1 | Chr1 | 19.5 |
| BD0145_4 | Chr1 | 19.5 |
| BD3195_2 | Chr1 | 19.5 |
| BD3195_1 | Chr1 | 19.5 |
| BD1774_1 | Chr1 | 19.5 |
| BD4130_2 | Chr1 | 23.5 |
| BD1531_1 | Chr1 | 23.7 |
| BD2840_1 | Chr1 | 23.7 |
| BD0237_2 | Chr1 | 26.9 |
| BD0390_1 | Chr1 | 26.9 |
| BD0389_4 | Chr1 | 30.5 |
| Locus | Chromosome | Genetic Position (cM) |
| BD3539_1 | Chr1 | 38.6 |
| BD4187_7 | Chr1 | 45.7 |
| BD0589_1 | Chr1 | 45.7 |
| BD3533_1 | Chr1 | 52.3 |
| BD4817_1 | Chr1 | 58.6 |
| BD3180_1 | Chr1 | 62.4 |
| BD0555_1 | Chr1 | 66.6 |
| BD4815_1 | Chr1 | 68.5 |
| BD2829_1 | Chr1 | 70.3 |
| BD1359_1 | Chr1 | 71 |
| BD2380_1 | Chr1 | 73.9 |
| BD3519_1 | Chr1 | 75.3 |
| BD3518_1 | Chr1 | 80.5 |
| BD1730_1 | Chr1 | 80.5 |
| BD3517_1 | Chr1 | 82.3 |
| BD1642_9 | Chr1 | 89 |
| BD3514_1 | Chr1 | 89 |
| BD2131_1 | Chr1 | 90.6 |
| BD3512_2 | Chr1 | 92.9 |
| BD3512_1 | Chr1 | 92.9 |
| BD3164_1 | Chr1 | 96.9 |
| BD2039_1 | Chr1 | 99.2 |
| BD3508_1 | Chr1 | 101 |
| BD2298_2 | Chr1 | 101.7 |
| BD4791_1 | Chr1 | 103.5 |
| BD3506_1 | Chr1 | 105.1 |
| BD1723_1 | Chr1 | 108.4 |
| BD1723_2 | Chr1 | 108.4 |
| BD1990_1 | Chr1 | 110.3 |
| BD0827_1 | Chr1 | 115.5 |
| BD3504_2 | Chr1 | 115.5 |
| BD3503_3 | Chr1 | 118.4 |
| BD3502_1 | Chr1 | 119 |
| BD1202_1 | Chr1 | 121.9 |
| BD0821_3 | Chr1 | 121.9 |
| BD1985_1 | Chr1 | 122.2 |
| BD1306_2 | Chr1 | 128.5 |
| BD3155_1 | Chr1 | 131.9 |
| BD3133_1 | Chr1 | 131.9 |
| Locus | Chromosome | Genetic Position (cM) |
| BD5073_1 | Chr1 | 136.6 |
| BD2330_3 | Chr1 | 137 |
| BD4004_4 | Chr1 | 137 |
| BD3051_3 | Chr1 | 137.3 |
| BD5097_1 | Chr1 | 137.7 |
| BD2232_1 | Chr1 | 142.1 |
| BD1922_1 | Chr1 | 142.3 |
| BD1920_2 | Chr1 | 148.9 |
| BD1920_1 | Chr1 | 148.9 |
| BD0200_8 | Chr1 | 148.9 |
| BD5075_2 | Chr1 | 155.4 |
| BD5092_2 | Chr1 | 155.5 |
| BD3107_1 | Chr1 | 156.2 |
| BD1674_1 | Chr1 | 159.6 |
| BD4493_1 | Chr1 | 163.6 |
| BD4034_1 | Chr1 | 167.4 |
| BD0718_1 | Chr1 | 167.4 |
| BD0266_1 | Chr1 | 167.4 |
| BD0267_2 | Chr1 | 171.2 |
| BD1154_1 | Chr1 | 172.5 |
| BD4224_2 | Chr1 | 174.8 |
| BD3222_10 | Chr1 | 180.5 |
| BD1100_2 | Chr1 | 181.8 |
| BD0069_2 | Chr1 | 182.4 |
| BD1470_2 | Chr1 | 189.3 |
| BD1193_3 | Chr1 | 189.3 |
| BD0750_4 | Chr1 | 189.9 |
| BD4045_1 | Chr1 | 189.9 |
| BD1743_1 | Chr1 | 195.5 |
| BD4047_1 | Chr1 | 196.1 |
| BD4049_1 | Chr1 | 198.8 |
| BD3606_1 | Chr1 | 198.8 |
| BD2025_1 | Chr1 | 201.3 |
| BD1853_2 | Chr1 | 202.6 |
| BD4050_1 | Chr1 | 202.6 |
| BD3607_1 | Chr1 | 202.6 |
| BD3609_1 | Chr1 | 205.1 |
| BD4054_1 | Chr1 | 210.5 |
| BD1979_1 | Chr1 | 214.3 |
| BD1719_1 | Chr1 | 214.3 |
| BD3613_1 | Chr1 | 216.3 |
| BD3613_4 | Chr1 | 216.3 |
| Locus | Chromosome | Genetic Position (cM) |
| BD1084_1 | Chr1 | 216.9 |
| BD4056_1 | Chr1 | 219.8 |
| BD3231_2 | Chr1 | 220.6 |
| BD2192_8 | Chr1 | 220.6 |
| BD3616_1 | Chr1 | 221.1 |
| BD0469_5 | Chr1 | 222.5 |
| BD4848_2 | Chr1 | 224.7 |
| BD0177_1 | Chr1 | 229.7 |
| BD2545_1 | Chr1 | 232.9 |
| BD1622_8 | Chr1 | 232.9 |
| BD4233_1 | Chr1 | 232.9 |
| BD4061_3 | Chr1 | 233.5 |
| BD4061_1 | Chr1 | 233.5 |
| BD4062_1 | Chr1 | 236.2 |
| BD4854_3 | Chr1 | 245.7 |
| BD1929_1 | Chr1 | 255.7 |
| BD3634_2 | Chr1 | 257.7 |
| BD4075_1 | Chr1 | 257.7 |
| BD0454_1 | Chr1 | 258 |
| BD4076_1 | Chr1 | 260.5 |
| BD2553_3 | Chr1 | 261.8 |
| BD4077_1 | Chr1 | 261.8 |
| BD3636_2 | Chr1 | 261.8 |
| BD3636_1 | Chr1 | 261.8 |
| BD1404_2 | Chr1 | 264.5 |
| BD2410_1 | Chr1 | 266 |
| BD1056_4 | Chr1 | 269 |
| BD4078_6 | Chr1 | 273.4 |
| BD3248_1 | Chr1 | 277.6 |
| BD1593_2 | Chr1 | 278.4 |
| BD4860_2 | Chr1 | 284.8 |
| BD1314_1 | Chr1 | 291.7 |
| BD3643_1 | Chr1 | 293.9 |
| BD0987_2 | Chr1 | 297.3 |
| BD1778_1 | Chr1 | 297.3 |
| BD0366_3 | Chr1 | 297.8 |
| BD4248_1 | Chr1 | 305.1 |
| BD1606_8 | Chr1 | 313.2 |
| BD0340_2 | Chr1 | 314.5 |
| BD3646_1 | Chr1 | 320.3 |
| BD0639_3 | Chr1 | 347.8 |
| BD2388_4 | Chr1 | 347.8 |
| Locus | Chromosome | Genetic Position (cM) |
| BD4254_2 | Chr1 | 347.8 |
| BD4907_1 | Chr2 | 0 |
| BD4907_6 | Chr2 | 0 |
| BD3719_1 | Chr2 | 21.2 |
| BD2595_6 | Chr2 | 27.7 |
| BD1569_2 | Chr2 | 42.8 |
| BD2915_1 | Chr2 | 43.7 |
| BD4293_1 | Chr2 | 58.4 |
| BD0284_1 | Chr2 | 58.4 |
| BD4900_1 | Chr2 | 63.8 |
| BD3711_1 | Chr2 | 66.8 |
| BD3711_2 | Chr2 | 66.8 |
| BD1663_1 | Chr2 | 67.5 |
| BD0156_2 | Chr2 | 85.8 |
| BD3286_1 | Chr2 | 88.8 |
| BD1368_10 | Chr2 | 89.6 |
| BD2123_1 | Chr2 | 90.4 |
| BD1460_3 | Chr2 | 91.9 |
| BD0494_2 | Chr2 | 96.7 |
| BD3700_1 | Chr2 | 104.2 |
| BD3699_1 | Chr2 | 105 |
| BD3698_4 | Chr2 | 108.8 |
| BD4282_1 | Chr2 | 115.5 |
| BD4891_1 | Chr2 | 115.7 |
| BD0164_1 | Chr2 | 118.2 |
| BD0893_1 | Chr2 | 120.3 |
| BD0893_2 | Chr2 | 120.3 |
| BD1266_1 | Chr2 | 121.1 |
| BD4279_1 | Chr2 | 124.4 |
| BD4275_1 | Chr2 | 132.1 |
| BD3277_1 | Chr2 | 136.5 |
| BD4273_1 | Chr2 | 137.2 |
| BD0634_3 | Chr2 | 138 |
| BD0635_1 | Chr2 | 138.3 |
| BD1586_3 | Chr2 | 139.4 |
| BD1268_2 | Chr2 | 142.6 |
| BD0946_1 | Chr2 | 148.6 |
| BD0661_1 | Chr2 | 148.6 |
| BD0501_3 | Chr2 | 148.6 |
| BD0399_5 | Chr2 | 149 |
| BD0194_5 | Chr2 | 149.4 |
| BD3666_2 | Chr2 | 149.9 |
| Locus | Chromosome | Genetic Position (cM) |
| BD1224_4 | Chr2 | 150.5 |
| BD3785_2 | Chr2 | 152.5 |
| BD2950_8 | Chr2 | 157.5 |
| BD3787_1 | Chr2 | 159.1 |
| BD0786_4 | Chr2 | 159.9 |
| BD3788_9 | Chr2 | 162.8 |
| BD4928_5 | Chr2 | 162.8 |
| BD3789_1 | Chr2 | 163.1 |
| BD4344_1 | Chr2 | 163.1 |
| BD1601_2 | Chr2 | 164.2 |
| BD2217_2 | Chr2 | 164.6 |
| BD3790_3 | Chr2 | 165.2 |
| BD3790_1 | Chr2 | 165.2 |
| BD0296_2 | Chr2 | 171.4 |
| BD0295_3 | Chr2 | 173.4 |
| BD4345_1 | Chr2 | 174.4 |
| BD3347_1 | Chr2 | 180.6 |
| BD4351_4 | Chr2 | 188.4 |
| BD4352_1 | Chr2 | 189.7 |
| BD0797_4 | Chr2 | 189.9 |
| BD4359_1 | Chr2 | 195.4 |
| BD4359_2 | Chr2 | 195.4 |
| BD0493_1 | Chr2 | 195.4 |
| BD4337_1 | Chr2 | 195.8 |
| BD0492_3 | Chr2 | 195.8 |
| BD0904_1 | Chr2 | 199 |
| BD0455_3 | Chr2 | 202.2 |
| BD0419_4 | Chr2 | 235.7 |
| BD1346_1 | Chr2 | 248.1 |
| BD3364_3 | Chr2 | 253.3 |
| BD2968_1 | Chr2 | 254.1 |
| BD0604_1 | Chr2 | 257.5 |
| BD3367_1 | Chr2 | 260.2 |
| BD3819_3 | Chr2 | 261.9 |
| BD3368_1 | Chr2 | 261.9 |
| BD3821_1 | Chr2 | 272.9 |
| BD0089_1 | Chr2 | 273.4 |
| BD0889_2 | Chr2 | 282.7 |
| BD3824_2 | Chr2 | 286.5 |
| BD3824_1 | Chr2 | 286.5 |
| BD2664_1 | Chr2 | 292.3 |
| BD0309_1 | Chr2 | 295.7 |
| Locus | Chromosome | Genetic Position (cM) |
| BD0953_6 | Chr2 | 295.7 |
| BD3827_1 | Chr2 | 299.5 |
| BD2181_2 | Chr2 | 301.6 |
| BD3371_1 | Chr2 | 308.8 |
| BD1374_1 | Chr2 | 357.9 |
| BD3101_1 | Chr2 | 361.7 |
| BD1938_1 | Chr2 | 367.8 |
| BD1939_1 | Chr2 | 376.8 |
| BD1379_2 | Chr2 | 381.2 |
| Bsr1 | Chr3 | 0 |
| BD3899_2 | Chr3 | 5.6 |
| BD4417_1 | Chr3 | 5.6 |
| BD0842_3 | Chr3 | 13 |
| BD3008_2 | Chr3 | 18.4 |
| BD1994_2 | Chr3 | 29.7 |
| BD1786_1 | Chr3 | 38 |
| BD2707_1 | Chr3 | 41.8 |
| BD0458_4 | Chr3 | 54.3 |
| BD0976_1 | Chr3 | 58.9 |
| BD0335_1 | Chr3 | 64.9 |
| BD2599_1 | Chr3 | 76.5 |
| BD1415_1 | Chr3 | 82.5 |
| BD1188_3 | Chr3 | 84.1 |
| BD0226_5 | Chr3 | 84.1 |
| BD1436_1 | Chr3 | 91.9 |
| BD1902_3 | Chr3 | 92.7 |
| BD3725_1 | Chr3 | 93.3 |
| BD1153_5 | Chr3 | 98.5 |
| BD2600_3 | Chr3 | 102.3 |
| BD4910_1 | Chr3 | 108.3 |
| BD3737_1 | Chr3 | 114 |
| BD3739_1 | Chr3 | 120.7 |
| BD3311_1 | Chr3 | 132.1 |
| BD0899_5 | Chr3 | 135.7 |
| BD1321_1 | Chr3 | 135.7 |
| BD4316_1 | Chr3 | 136.4 |
| BD3314_2 | Chr3 | 143.7 |
| BD2239_2 | Chr3 | 146.2 |
| BD2239_1 | Chr3 | 146.5 |
| BD2286_1 | Chr3 | 147.8 |
| BD3746_2 | Chr3 | 147.8 |
| BD1713_8 | Chr3 | 149.1 |
| Locus | Chromosome | Genetic Position (cM) |
| BD2133_1 | Chr3 | 150.6 |
| BD3750_1 | Chr3 | 151.2 |
| BD3750_2 | Chr3 | 151.2 |
| BD1265_3 | Chr3 | 151.5 |
| BD0174_9 | Chr3 | 156.3 |
| BD0811_1 | Chr3 | 156.3 |
| BD2618_3 | Chr3 | 156.3 |
| BD1009_2 | Chr3 | 157.8 |
| BD2384_1 | Chr3 | 157.8 |
| BD3754_1 | Chr3 | 158.7 |
| BD2933_1 | Chr3 | 160.7 |
| BD1111_1 | Chr3 | 161.7 |
| BD0590_1 | Chr3 | 162.5 |
| BD3755_1 | Chr3 | 163.8 |
| BD0371_1 | Chr3 | 166.3 |
| BD0790_5 | Chr3 | 167.9 |
| BD0756_1 | Chr3 | 168.2 |
| BD3758_5 | Chr3 | 168.2 |
| BD3759_1 | Chr3 | 168.2 |
| BD1381_1 | Chr3 | 168.2 |
| BD1742_2 | Chr3 | 171.9 |
| BD0507_4 | Chr3 | 173.4 |
| BD1141_7 | Chr3 | 174.4 |
| BD0809_2 | Chr3 | 175.2 |
| BD2073_1 | Chr3 | 175.2 |
| BD2073_2 | Chr3 | 175.2 |
| BD3959_1 | Chr3 | 175.4 |
| BD1904_1 | Chr3 | 175.9 |
| BD1627_1 | Chr3 | 177 |
| BD2474_1 | Chr3 | 177.3 |
| BD3958_11 | Chr3 | 178.9 |
| BD0741_1 | Chr3 | 179.3 |
| BD0742_1 | Chr3 | 179.7 |
| BD0859_4 | Chr3 | 180.1 |
| BD2241_1 | Chr3 | 185.6 |
| BD2731_2 | Chr3 | 189.3 |
| BD3946_14 | Chr3 | 189.7 |
| BD0926_1 | Chr3 | 189.7 |
| BD4445_9 | Chr3 | 194.1 |
| BD4445_11 | Chr3 | 194.1 |
| BD0193_8 | Chr3 | 196.6 |
| BD2080_4 | Chr3 | 200.8 |
| Locus | Chromosome | Genetic Position (cM) |
| BD0192_6 | Chr3 | 203.7 |
| BD1637_3 | Chr3 | 203.7 |
| BD3425_4 | Chr3 | 215 |
| BD3425_3 | Chr3 | 215 |
| BD1855_2 | Chr3 | 215 |
| BD1521_3 | Chr3 | 215 |
| BD0172_4 | Chr3 | 215.3 |
| BD0985_2 | Chr3 | 218.4 |
| BD3939_1 | Chr3 | 223.3 |
| BD3027_3 | Chr3 | 224 |
| BD1618_1 | Chr3 | 224.3 |
| BD1130_1 | Chr3 | 224.3 |
| BD0836_1 | Chr3 | 226.4 |
| BD4401_10 | Chr3 | 227.1 |
| BD2686_1 | Chr3 | 233.3 |
| BD1964_2 | Chr3 | 238.7 |
| BD1964_1 | Chr3 | 238.7 |
| BD1497_1 | Chr3 | 243.7 |
| BD0277_1 | Chr3 | 248.7 |
| BD4963_5 | Chr3 | 262.2 |
| BD0182_2 | Chr3 | 265.6 |
| BD2323_9 | Chr3 | 265.9 |
| BD1806_3 | Chr3 | 267.2 |
| BD0421_4 | Chr3 | 267.2 |
| BD0421_3 | Chr3 | 267.2 |
| BD0181_1 | Chr3 | 269.9 |
| BD1129_1 | Chr3 | 280.8 |
| BD1882_4 | Chr3 | 281.4 |
| BD0303_1 | Chr3 | 283.7 |
| BD0303_2 | Chr3 | 283.7 |
| BD0881_2 | Chr3 | 288.3 |
| BD3379_5 | Chr3 | 290.5 |
| BD3853_1 | Chr3 | 293 |
| BD1050_2 | Chr3 | 295.1 |
| BD0695_5 | Chr3 | 295.9 |
| BD3852_2 | Chr3 | 301.9 |
| BD1914_3 | Chr3 | 313.4 |
| BD0271_3 | Chr3 | 316.4 |
| BD1915_2 | Chr3 | 316.4 |
| BD4388_1 | Chr3 | 317.2 |
| BD2978_2 | Chr3 | 318.3 |
| BD3376_1 | Chr3 | 318.3 |
| Locus | Chromosome | Genetic Position (cM) |
| BD1157_1 | Chr3 | 322.7 |
| BD1482_1 | Chr3 | 325.6 |
| BD2475_1 | Chr3 | 330.8 |
| BD0378_2 | Chr3 | 338.1 |
| BD4959_1 | Chr3 | 343.3 |
| BD3843_6 | Chr3 | 344.4 |
| BD4958_1 | Chr3 | 345.7 |
| BD3837_2 | Chr3 | 372.2 |
| BD4954_3 | Chr3 | 374.4 |
| BD4953_1 | Chr3 | 378.4 |
| BD2975_1 | Chr3 | 384.8 |
| BD4379_1 | Chr3 | 385.5 |
| BD4952_1 | Chr3 | 391.9 |
| BD3832_1 | Chr3 | 397.1 |
| BD2973_10 | Chr3 | 404.5 |
| BD3831_6 | Chr3 | 407.3 |
| BD4377_2 | Chr3 | 408 |
| BD0955_1 | Chr3 | 413 |
| BD3830_1 | Chr3 | 417 |
| BD0888_2 | Chr3 | 418.6 |
| BD1418_2 | Chr4 | 0 |
| BD2019_1 | Chr4 | 10.6 |
| BD1271_1 | Chr4 | 19.2 |
| BD3052_6 | Chr4 | 28.3 |
| BD0248_1 | Chr4 | 32.3 |
| BD0465_1 | Chr4 | 50.8 |
| BD1016_1 | Chr4 | 52.9 |
| BD1248_1 | Chr4 | 60.1 |
| BD0922_2 | Chr4 | 63.5 |
| BD1309_2 | Chr4 | 74.9 |
| BD0498_2 | Chr4 | 77.6 |
| BD3092_16 | Chr4 | 82.8 |
| BD4022_1 | Chr4 | 82.8 |
| BD4023_2 | Chr4 | 84.9 |
| BD4023_3 | Chr4 | 84.9 |
| BD3097_1 | Chr4 | 95.4 |
| BD2462_1 | Chr4 | 96.2 |
| BD2156_1 | Chr4 | 96.7 |
| BD4411_1 | Chr4 | 97.9 |
| BD0087_2 | Chr4 | 98.5 |
| BD0087_1 | Chr4 | 98.5 |
| BD3892_3 | Chr4 | 101.5 |
| Locus | Chromosome | Genetic Position (cM) |
| BD3892_1 | Chr4 | 101.5 |
| BD1540_1 | Chr4 | 101.5 |
| BD1291_1 | Chr4 | 102.5 |
| BD0231_4 | Chr4 | 103.3 |
| BD1416_5 | Chr4 | 103.3 |
| BD0212_16 | Chr4 | 103.3 |
| BD2473_2 | Chr4 | 104.3 |
| BD0450_1 | Chr4 | 104.3 |
| BD2705_5 | Chr4 | 106.8 |
| BD1856_5 | Chr4 | 108.1 |
| BD3005_6 | Chr4 | 108.4 |
| BD1658_3 | Chr4 | 110.2 |
| BD0427_4 | Chr4 | 111 |
| BD2704_1 | Chr4 | 111 |
| BD3890_2 | Chr4 | 111 |
| BD1866_1 | Chr4 | 111 |
| BD1361_2 | Chr4 | 111.6 |
| BD3004_2 | Chr4 | 114 |
| BD3889_3 | Chr4 | 115.8 |
| BD4980_4 | Chr4 | 115.8 |
| BD0460_1 | Chr4 | 115.8 |
| BD2177_4 | Chr4 | 117.6 |
| BD3887_1 | Chr4 | 118.1 |
| BD3886_1 | Chr4 | 118.7 |
| BD1073_1 | Chr4 | 122 |
| BD0833_2 | Chr4 | 127.3 |
| BD3399_2 | Chr4 | 130.2 |
| BD3399_1 | Chr4 | 130.2 |
| BD4975_1 | Chr4 | 130.8 |
| BD3876_1 | Chr4 | 130.8 |
| BD1893_1 | Chr4 | 136.8 |
| BD3585_2 | Chr4 | 137.3 |
| BD0521_3 | Chr4 | 137.3 |
| BD1067_1 | Chr4 | 137.3 |
| BD0258_4 | Chr4 | 137.3 |
| BD1367_1 | Chr4 | 137.3 |
| BD3589_1 | Chr4 | 138.4 |
| BD4842_1 | Chr4 | 138.4 |
| BD3591_2 | Chr4 | 139.7 |
| BD3591_1 | Chr4 | 139.7 |
| BD1469_1 | Chr4 | 139.7 |
| BD4222_1 | Chr4 | 141.7 |
| Locus | Chromosome | Genetic Position (cM) |
| BD0180_10 | Chr4 | 143.2 |
| BD0342_5 | Chr4 | 143.8 |
| BD3595_2 | Chr4 | 144.8 |
| BD4044_5 | Chr4 | 145.1 |
| BD0013_2 | Chr4 | 145.4 |
| BD3596_1 | Chr4 | 145.4 |
| BD2534_2 | Chr4 | 146.2 |
| BD2058_1 | Chr4 | 150.2 |
| BD2071_1 | Chr4 | 150.5 |
| BD0311_1 | Chr4 | 150.8 |
| BD2057_1 | Chr4 | 150.8 |
| BD2451_2 | Chr4 | 151.1 |
| BD5063_2 | Chr4 | 152.6 |
| BD1105_1 | Chr4 | 154.6 |
| BD1104_1 | Chr4 | 154.6 |
| BD3938_2 | Chr4 | 157.1 |
| BD1944_1 | Chr4 | 157.1 |
| BD0525_3 | Chr4 | 157.7 |
| BD5004_2 | Chr4 | 158.5 |
| BD5004_1 | Chr4 | 158.5 |
| BD0984_6 | Chr4 | 161.6 |
| BD0522_2 | Chr4 | 162.4 |
| BD2722_1 | Chr4 | 165.8 |
| BD0574_2 | Chr4 | 167.9 |
| BD5001_3 | Chr4 | 171.9 |
| BD3925_1 | Chr4 | 177.7 |
| BD3922_1 | Chr4 | 180.2 |
| BD0417_9 | Chr4 | 199.7 |
| BD2346_1 | Chr4 | 207.9 |
| BD0418_1 | Chr4 | 210.5 |
| BD0748_1 | Chr4 | 213 |
| BD3912_2 | Chr4 | 213 |
| BD1582_1 | Chr4 | 220.1 |
| BD0690_3 | Chr4 | 222.9 |
| BD1840_1 | Chr4 | 233.7 |
| BD0404_2 | Chr4 | 241.5 |
| BD0998_1 | Chr4 | 259.7 |
| BD3902_1 | Chr4 | 275.9 |
| BD0382_1 | Chr4 | 288.6 |
| BD3901_1 | Chr4 | 292.4 |
| BD0676_1 | Chr4 | 297.4 |
| BD0348_1 | Chr4 | 297.4 |
| Locus | Chromosome | Genetic Position (cM) |
| BD4986_1 | Chr4 | 301.5 |
| BD1794_3 | Chr4 | 304.4 |
| BD1794_1 | Chr4 | 304.4 |
| BD0021_2 | Chr4 | 304.7 |
| BD2636_1 | Chr5 | 0 |
| BD0114_3 | Chr5 | 7.8 |
| BD0289_1 | Chr5 | 25.2 |
| BD2635_1 | Chr5 | 25.2 |
| BD0471_3 | Chr5 | 45.4 |
| BD0175_2 | Chr5 | 47.3 |
| BD2633_1 | Chr5 | 47.3 |
| BD0036_2 | Chr5 | 48.8 |
| BD0356_8 | Chr5 | 48.8 |
| BD4921_1 | Chr5 | 49.4 |
| BD1139_1 | Chr5 | 49.4 |
| BD2438_1 | Chr5 | 49.4 |
| BD3329_2 | Chr5 | 49.4 |
| BD3329_3 | Chr5 | 49.4 |
| BD2435_1 | Chr5 | 49.4 |
| BD1107_1 | Chr5 | 49.4 |
| BD4326_1 | Chr5 | 49.4 |
| BD3767_1 | Chr5 | 49.4 |
| BD3262_1 | Chr5 | 49.9 |
| BD3660_1 | Chr5 | 50.6 |
| BD3260_5 | Chr5 | 50.9 |
| BD2563_1 | Chr5 | 51.2 |
| BD0024_1 | Chr5 | 51.2 |
| BD4261_2 | Chr5 | 51.5 |
| BD2094_2 | Chr5 | 51.5 |
| BD3982_7 | Chr5 | 51.5 |
| BD1798_1 | Chr5 | 51.7 |
| BD3052_2 | Chr5 | 51.8 |
| BD0220_1 | Chr5 | 52.6 |
| BD0637_1 | Chr5 | 56.6 |
| BD3988_1 | Chr5 | 57.4 |
| BD2754_3 | Chr5 | 58.4 |
|  |  |  |
| Locus | Chromosome | Genetic Position (cM) |
| BD0077_3 | Chr5 | 58.4 |
| BD3061_1 | Chr5 | 58.4 |
| BD0173_2 | Chr5 | 58.4 |
| BD3999_1 | Chr5 | 67.3 |
| BD3999_2 | Chr5 | 67.3 |
| BD4001_2 | Chr5 | 68.8 |
| BD4476_1 | Chr5 | 69.6 |
| BD2291_3 | Chr5 | 72.1 |
| BD0783_1 | Chr5 | 72.1 |
| BD1967_1 | Chr5 | 72.4 |
| BD5021_1 | Chr5 | 73.4 |
| BD1199_1 | Chr5 | 75.4 |
| BD1950_1 | Chr5 | 79 |
| BD1950_2 | Chr5 | 79 |
| BD0761_1 | Chr5 | 90.7 |
| BD3447_3 | Chr5 | 113 |
| BD0218_4 | Chr5 | 113.3 |
| BD1641_1 | Chr5 | 116.2 |
| BD0149_1 | Chr5 | 118.5 |
| BD3974_1 | Chr5 | 121.4 |
| BD0141_1 | Chr5 | 122 |
| BD3448_2 | Chr5 | 126.4 |
| BD4464_1 | Chr5 | 133.6 |
| BD0482_1 | Chr5 | 144.4 |
| BD4088_3 | Chr5 | 153.9 |
| BD4088_6 | Chr5 | 153.9 |
| BD1676_1 | Chr5 | 155 |
| BD3488_1 | Chr5 | 165.1 |
| BD0115_3 | Chr5 | 171.5 |
| BD0213_1 | Chr5 | 172.5 |
| BD1078_3 | Chr5 | 175.2 |
| BD4159_1 | Chr5 | 185.5 |
| BD0434_2 | Chr5 | 190.7 |
| BD2811_2 | Chr5 | 193.5 |
| BD2811_3 | Chr5 | 193.5 |
| BD0868_1 | Chr5 | 197.3 |
| BD0607_1 | Chr5 | 198 |
